# Vector competence of *Aedes albopictus* populations for chikungunya virus is shaped by their demographic history

**DOI:** 10.1038/s42003-020-1046-6

**Published:** 2020-06-24

**Authors:** Anubis Vega-Rúa, Michele Marconcini, Yoann Madec, Mosè Manni, Davide Carraretto, Ludvik Marcus Gomulski, Giuliano Gasperi, Anna-Bella Failloux, Anna Rodolfa Malacrida

**Affiliations:** 1 Laboratory of Vector Control Research, Institut Pasteur of Guadeloupe, 97139 Guadeloupe, France; 2 Department of Biology and Biotechnology, University of Pavia, Via Ferrata 9, 27100 Pavia, Italy; 3 Department of Infection and Epidemiology of Emerging Diseases, Institut Pasteur, 25-28 rue du Dr Roux, 75724 Paris, France; 4 Department of Genetic Medicine and Development, University of Geneva Medical School, 1 rue Michel-Servet 1211 Genève and Swiss Institute of Bioinformatics, Geneva, Switzerland; 5 Department of Virology, Arboviruses and Insect Vectors Unit, Institut Pasteur, 25-28 rue du Dr Roux, 75724 Paris, France

**Keywords:** Entomology, Genetic variation

## Abstract

The mosquito *Aedes albopictus* is one of the most dangerous invasive species. Its worldwide spread has created health concerns as it is a major vector of arboviruses of public health significance such as chikungunya (CHIKV). Dynamics of different genetic backgrounds and admixture events may have impacted competence for CHIKV in adventive populations. Using microsatellites, we infer the genetic structure of populations across the expansion areas that we then associate with their competence for different CHIKV genotypes. Here we show that the demographic history of *Ae. albopictus* populations is a consequence of rapid complex patterns of historical lineage diversification and divergence that influenced their competence for CHIKV. The history of adventive populations is associated with CHIKV genotypes in a genotype-by-genotype interaction that impacts their vector competence. Thus, knowledge of the demographic history and vector competence of invasive mosquitoes is pivotal for assessing the risk of arbovirus outbreaks in newly colonized areas.

## Abstract (web summary)

Anubis Vega-Rúa, Michele Marconcini et al. study how the dynamics of genetics and admixture events of *Aedes albopictus* affect its competence for the CHIKV. Using microsatellites, they found that the demographic history influences the competence for the virus and is an important feature to assess the risk of outbreaks in new areas.

## Introduction

Understanding the genetic processes associated with species introduction and establishment in new environments is fundamental to infer the evolutionary mechanisms underlying the dynamics and history of invasive processes. But, when an invasive species is a threat to human health, such as a disease vector, knowledge of population dynamics and demography is useful to interpret the causes and predict the risks of disease outbreaks. *Aedes albopictus* (Skuse 1894), the Asian tiger mosquito, is an emblematic example of an arbovirus vector that has rapidly and successfully invaded much of the globe supported by its life history traits and high propagule pressure driven by human activities. This mosquito took four decades to invade the world, while its counterpart *Aedes aegypti* did so in four centuries^1^. From its home range in tropical Southeast Asia, where it was originally a zoophilic forest species^2^, *Ae. albopictus* spread first to the islands in the Indian and Pacific Oceans^3^ and, during the 1980s, rapidly extended its range across temperate regions in Europe, the Americas and Africa^1,4^. This mosquito is able to tolerate climate/environment interactions that differ from its home range^5–7^. Its ability to diapause during unfavourable seasons and to lay desiccation-resistant eggs has facilitated its expansion in temperate regions^8–11^. It is listed among the top hundred most dangerous invasive species identified^12^. Its spread has created public health concerns as experimental infections have shown the species’ competence for at least 20 arboviruses and it is considered the main vector for chikungunya (CHIKV), and to a lesser degree for dengue (DENV) and Zika (ZIKV) viruses^13–21^. Anthropogenic activities, by creating new breeding and trophic niches of adaptation close to human habitations^22,23^, strengthened the association of *Ae. albopictus* with humans, with important public health implications. Indeed, beyond its original range, it has been increasingly involved in local autochthonous transmission of chikungunya in many places where it has become established, including La Réunion, continental Europe, Africa, the Americas and Japan, determining major CHIKV epidemics of severe, persistent, debilitating arthralgia^24–27^. Chikungunya virus (CHIKV, genus Alphavirus in the family Togaviridae), first discovered in 1952 in Tanzania^28^ and generally transmitted by *Aedes* mosquitoes, comprises three genetic lineages: Asian, West African and East/Central/South African (ECSA) which apparently evolved independently in different geographic areas^29^. The fact that *Ae. albopictus* is becoming the major vector of CHIKV is due to a series of adaptive mutations in the CHIKV ECSA lineage, the most known being an alanine to valine substitution at position 226 of the E1 glycoprotein (ECSA E1-A226V). This adaptive mutation, established in the Indian Ocean region in 2004–2005, dramatically increased the vector competence of *Ae. albopictus* for CHIKV, with respect to the previously more typical vector, *Ae. aegypti*. In association with the high invasive potential of *Ae. albopictus*, the ECSA E1-226V variant rapidly spread even in temperate areas such as Europe, causing CHIKV outbreaks.

The identification of the main dispersal routes of this mosquito vector can help in mitigating its spread and preventing/reducing the risks of arboviral outbreaks as once a vector is introduced, autochthonous transmission of its associated arboviruses may rapidly follow. But this implies knowledge of the demographic history of invading populations, their degree of connectivity and their vectorial capacities^7^. Several genetic, ecological and behavioural studies were performed to unravel different aspects of the invasion process and to infer the degree of genetic connectivity among populations at the micro- and macro-geographic levels^23,30–36^. However, vector competence for CHIKV has been found to be highly variable within and between populations, with transmission efficiencies that are strictly dependent on specific combinations of mosquito genome, viral genetic characteristics and temperature in a genotype-by-genotype-by-environment interaction^37–39^. On this basis, we previously proposed that admixture events and dynamics of different genetic backgrounds during the invasion process of this mosquito may have impacted competence for CHIKV in adventive populations^32^. In this study, we tested this hypothesis. We first provided a picture of the major events that determined, at the macrogeographic level, the demographic history of the adventive populations out of Southeast Asia starting from the 18th century. Secondly, we notably found that the population demographic history impacted their vector competence for CHIKV. We have demonstrated that the demographic history of populations was tightly related to CHIKV genotypes in a sort of adaptive coevolution, where genotype-by-genotype interactions influenced the corresponding *Ae. albopictus* vector competence phenotypes for this virus.

## Results

### Proximity does not impact population genetic connectivity

**Fig. 1:**
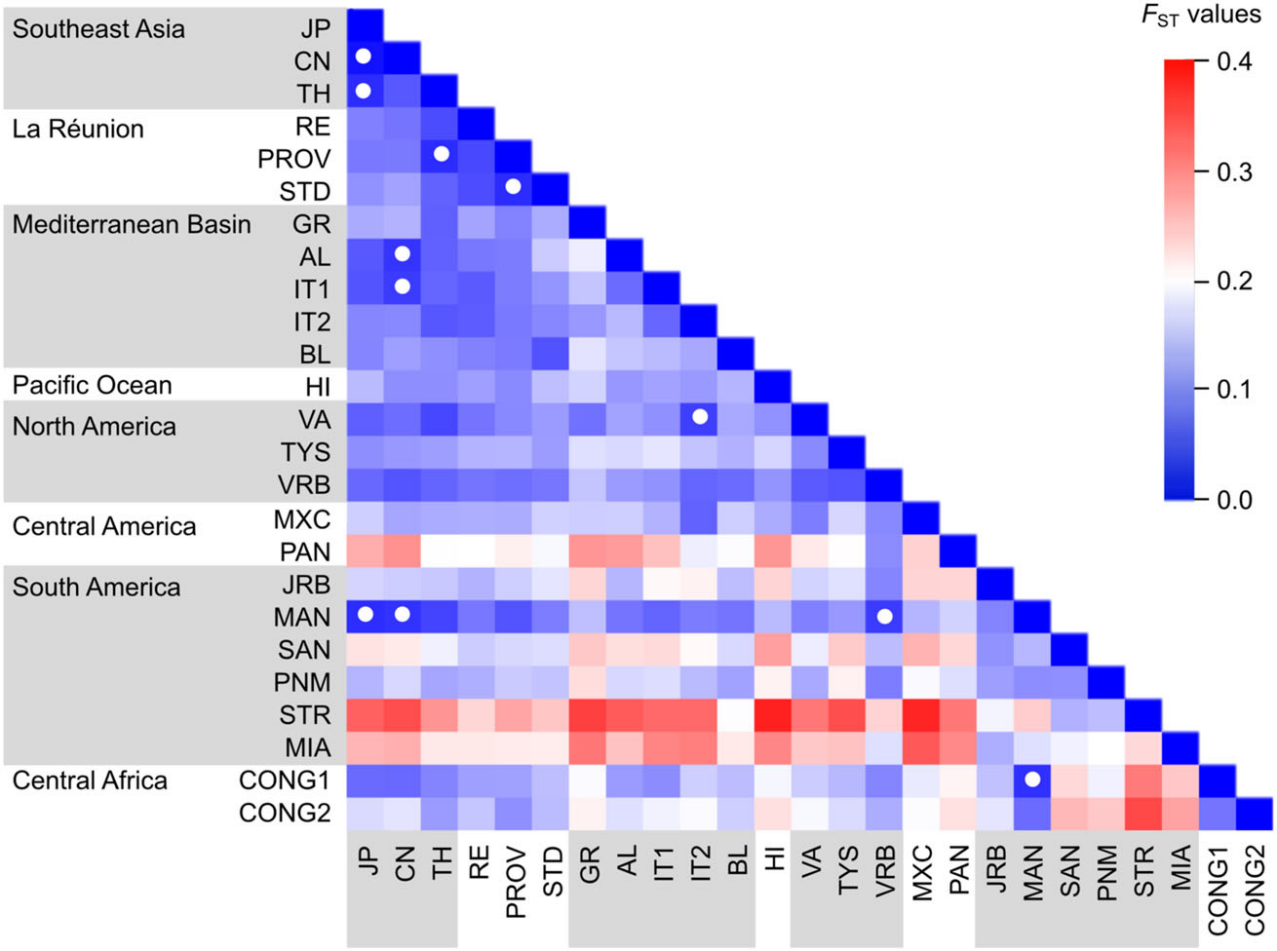
Matrix of pairwise *F*_ST_ values among 25 *Ae. albopictus* population samples. *F*_ST_ values highlighted with a white circle are not significantly different from zero (*P* > 0.05) after Bonferroni correction.

AMOVA analyses of molecular variance across mosquitoes from 25 localities spanning from Asia to Mediterranean area, the Americas and Central Africa (Table 1), indicated that 83% of variance occurred within populations, while only 11% and 6% were detected between populations and regions, respectively. The highest level of within-population variability was detected in the ancestral Southeast Asia region, where China displays the highest number of alleles and private alleles/individual. Outside this area, the adventive populations displayed different degrees of variability erosion (Supplementary Table 1). No signs of isolation by distance (IBD) were detected globally (*y* = 1e−06× + 0.0805; *R*^2^ = 0.015) nor within regions (*R*^2^ = 0.023 in South America, *R*^2^ = 0.15 in the Mediterranean area). Moreover, the population differentiation has been found to be heterogeneous not only between but also within geographical regions (Fig. 1, Supplementary Table 2). The most homogeneous area, in terms of non-significant or very low *F*_ST_ values, was the ancestral Southeast Asia (*F*_ST_ 0.014 – 0.069), which maintains low levels of differentiation with La Réunion and the Mediterranean Basin (*F*_ST_ 0.041 – 0.140), and with North America (*F*_ST_ 0.056 – 0.124). The Mediterranean area appears to be heterogeneous (*F*_ST_ 0.081 – 0.186). Within North America, Florida (VRB) was the least differentiated population (*F*_ST_ 0.065 − 0.070), sharing connectivity with the populations from Asia, La Réunion, and the Mediterranean area. It was the only population that maintained genetic connectivity with Central and South America. These latter two regions were the most heterogeneous based on *F*_ST_ values. The Brazilian and Argentinian populations had no genetic affinities with those from Southeast Asia, the Mediterranean area or North America, with the exception of Manaus (MAN) in the Brazilian State of Amazonas that, in turn, maintained low levels of genetic differentiation with the populations from Congo in Africa.

**Table 1:** *Aedes albopictus* populations sampled in areas in which the presence of this mosquito has been historically documented.

Region	Country	1st historical record	Population	Population code	Sample size^a^	Latitude	Longitude	Date of collection
Southeast Asia	Japan	–	Nagasaki	JP	13	32.75°	129.88°	2011
	China	-	Xiamen	CN	10	24.47°	118.09°	2011
	Thailand	–	Ban Rai	TH	30	15.30°	99.45°	2010
Indian Ocean	La Réunion	18th century^41^	St Pierre	RE	30	−21.32°	55.47°	2010
			Providence	PROV	30	−20.88°	55.45°	2010
			St André	STD	30	−20.97°	55.65°	2010
Mediterranean Basin	Greece	2003^17^	Athens	GR	29	37.98°	23.73°	2011
	Albania	1979^92^	Tirana	AL	24	41.33°	19.83°	2011
	Italy	1990^93^	Cesena^b^	IT1	31	44.14°	12.25°	2010
			Brescia	IT2	26	45.54°	10.22°	2010
	France	2004^94^	Bar sur Loup^b^	BL	30	43.70°	6.99°	2013
Pacific Ocean	Hawaii	1895^95^	Oahu	HI	29	21.43°	−158.00°	2012
North America	U.S.A.	1985^40^	Manassas (Virginia)	VA	30	38.75°	−77.47°	2012
		1986^96^	St Louis (Missouri)^b^	TYS	30	38.52°	−90.55°	2012
		1986^97^	Vero Beach (Florida)^b^	VRB	30	27.58°	−80.37°	2012
Central America	Mexico	2002^44^	Tapachula^b^	MXC	30	14.88°	−92.25°	2012
	Panama	2004^45^	Colón^b^	PAN	30	9.35°	−79.88°	2012
South America	Brazil	1986^98^	Jurujuba (Rio de Janeiro)^b^	JRB	30	−22.91°	−43.12°	2012
		2002^99^	Manaus (Amazonas)^b^	MAN	30	−3.10°	−60.05°	2012
		1986^100^	Santos (São Paulo)^b^	SAN	30	−23.95°	−46.33°	2012
		1986^100^	Parnamirim (Rio Grande do Norte)^b^	PNM	30	−5.90°	−35.27°	2012
		2002^100^	Santarém (Parà)^b^	STR	30	−2.42°	−54.70°	2012
	Argentina	1998^101^	Eldorado (Misiones)^b^	MIA	30	−25.60°	−54.56°	2012
Central Africa	Congo	2011^102^	Brazzaville^b^	CONG1	30	−4.27°	15.22°	2011
		2011^102^	Mfilou	CONG2	30	−4.27°	15.28°	2011

^a^Sample size refers to that used for microsatellite analyses.

^b^Population samples evaluated for vector competence for CHIKV strains.

### Different lineages are present across the invaded areas

**Fig. 2:**
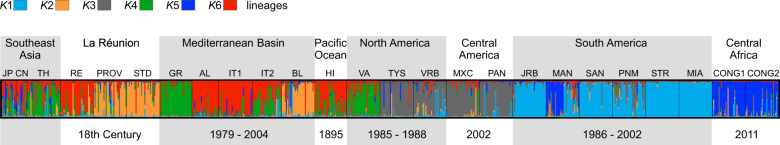
Representation of the co-ancestry of *Ae. albopictus* mosquitoes from the ancestral and derived regions. Dates of invasion in the different regions are shown in the lower part of the figure.

Six clusters/lineages represent the most parsimonious partitioning of the ancestry among individuals from the 25 populations (*K*1–*K*6 in Table 2) displaying a clear geographic distribution (Fig. 2, Supplementary Data 1). The genomes from the ancestral Asian populations, China and Japan, were distributed differently across clusters, but display the highest ancestry in *K*6. The other Asian population, Thailand, was fragmented mainly between *K*4 (55%) and *K*6 (24%). The ancestry heterogeneity of La Réunion and Mediterranean populations was obvious. The La Réunion mosquitoes from the southwest coast (RE) were mainly structured between *K*6 and *K*4 together with the majority of Mediterranean samples. By contrast, the two La Réunion populations from the north-eastern part (PROV and STD) were clustered with the French Mediterranean sample (BL) in *K*2. Hawaii was also fragmented between *K*6 and *K*4. North America appeared as a transition area in which populations such as Virginia (VA) still maintained membership in the two Asian clusters *K*4 (63%) and *K*6 (23%), while others such as Florida (VRB) and Missouri (TYS) exhibited ancestry profiles that transitioned to those typical of Central America (*K*3) and South America (*K*1). The Brazilian Amazonian (MAN) was fragmented across *K*1 (24%), *K*3 (17%) and notably the Southeast Asian *K*5 (45%). It is noteworthy that high percentages of *K*5 outside of Asia were recovered only in Manaus and in Congo, Africa (79%).

**Table 2:** Average coefficient of ancestry obtained from a STRUCTURE run with *K* = 6 for 702 individuals of *Ae. albopictus* from 25 samples collected in eight different geographical areas.

Region	Sample	*K*1	*K*2	*K*3	*K*4	*K*5	*K*6
Southeast Asia	JP	0.056	0.030	0.100	0.250	0.192	**0.371**
	CN	0.039	0.016	0.157	0.141	0.192	**0.455**
	TH	0.058	0.062	0.033	**0.546**	0.064	0.237
Indian Ocean	RE	0.045	0.120	0.019	0.113	0.013	**0.689**
	PROV	0.052	**0.621**	0.067	0.110	0.046	0.104
	STD	0.028	**0.782**	0.057	0.044	0.015	0.073
Mediterranean	GR	0.009	0.014	0.012	**0.854**	0.009	0.102
Basin	AL	0.024	0.010	0.033	0.077	0.033	**0.822**
	IT1	0.010	0.014	0.030	0.087	0.064	**0.795**
	IT2	0.053	0.033	0.105	0.347	0.009	**0.452**
	BL	0.074	**0.652**	0.097	0.027	0.053	0.096
Pacific Ocean	HI	0.013	0.020	0.056	0.268	0.015	**0.628**
North America	VA	0.022	0.014	0.084	**0.627**	0.020	0.232
	TYS	0.021	0.038	**0.804**	0.015	0.084	0.038
	VRB	0.148	0.061	**0.674**	0.020	0.038	0.060
Central America	MXC	0.009	0.052	**0.771**	0.056	0.025	0.086
	PAN	0.100	0.013	**0.841**	0.007	0.026	0.013
South America	JRB	**0.873**	0.011	0.065	0.009	0.027	0.015
	MAN	0.240	0.057	0.167	0.020	**0.450**	0.065
	SAN	**0.881**	0.028	0.057	0.010	0.010	0.014
	PNM	**0.600**	0.041	0.277	0.025	0.026	0.030
	STR	**0.960**	0.011	0.010	0.005	0.007	0.006
	MIA	**0.955**	0.010	0.011	0.006	0.011	0.007
Central Africa	CONG1	0.046	0.058	0.072	0.010	**0.792**	0.021
	CONG2	0.085	0.034	0.074	0.020	**0.762**	0.025

The highest value of coancestry of each population is highlighted in bold.

### Dynamics of invasion at the macrogeographic level

**Fig. 3:**
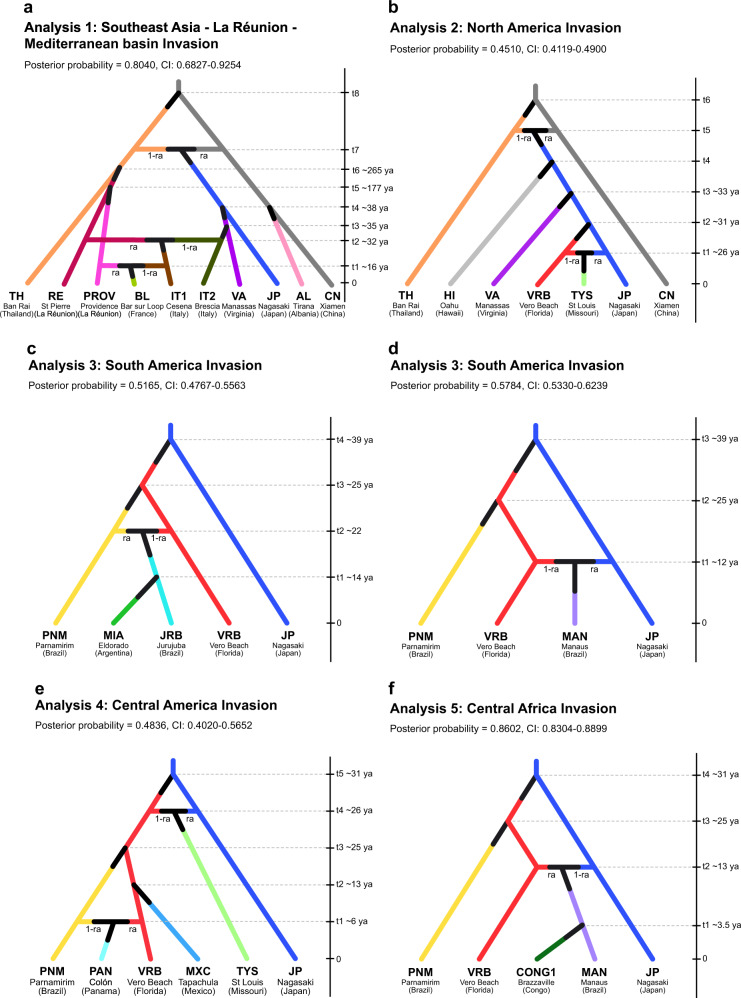
Graphical representation of the most-likely scenario of each set of scenarios describing the dynamics of samples within the native and derived areas using ABC methods. **a** Analysis 1: Southeast Asia, La Réunion and the Mediterranean invasion. **b** Analysis 2: The North America invasion. **c** Analysis 3: The South America Invasion. **d** The South America Invasion. **e** Analysis 4: Central American Invasion. **f** The Central Africa invasion.

Five separate sequential Bayesian ABC analyses (Supplementary Table 3, Fig. 3) were compared to acquire a macro geographic picture of the demographic history of invasive populations out of the Southeast Asian home range to La Réunion, to Mediterranean Basin, to Americas and Africa. In the ancestral area, China has been confirmed as the ancestral population from which Thailand diverged, while Japan emerged from an admixture between China and Thailand.

#### Out of Southeast Asia to La Réunion and to Mediterranean basin (analysis 1b, Fig. 3a)

Mosquitoes from the southwest coast of La Réunion, St Pierre (RE), have previously been suggested as having an East Asian derivation^32^. We assessed whether the colonization of southern and northern parts of the island occurred independently as direct events from external South Asian source areas or via secondary internal derivation events (analyses 1a). The direct derivation of the northern PROV from the south-western RE was the most supported hypothesis. The STD population appeared to have a similar derivation. Manni and colleagues^32^ suggested that La Réunion/St Pierre (RE) contributed to the origin of the North Italian IT1 population (Cesena) through an admixture event with another North Italian population IT2, (previously derived from the North American Virginia (VA) population). On this basis, we tested whether La Réunion contributed also to the establishment of another Mediterranean population, Bar-sur-Loup (BL) in southern France. The hypothesis that La Réunion (PROV) in admixture events (74%) with the Italian IT1 (26%) contributed to the French Bar-sur-Loup population was strongly supported.

#### North America invasion

The South Atlantic states such as Florida and Virginia and the Midwestern state of Missouri are considered the first states to be invaded by *Ae. albopictus* according to historical records (Table 1). Thus, Manassas (VA) in Virginia, Vero Beach (VRB) in Florida and St Louis (TYS) in Missouri were considered as representative populations of these first invasions. After reconfirming that VA was derived from Japan (JP), the origin of Florida/VRB was determined. The most statistically supported scenario indicated that VRB in Florida was also a derivation of JP (analysis 2a, Fig. 3b). As for St Louis (TYS) in Missouri (analysis 2b), the strongest scenario supported its derivation from an admixture between JP and Florida (VRB) (66% and 34% respectively).

#### South America

The analysis of this area was considered before that of Central America, where the documented presence of this mosquito was more recent (Table 1). The tested hypothesis (analyses 3a–d, Fig. 3c, d) was whether the demographic histories of South American populations were related to those of North America, considering the concomitant historical presence of the species. Indeed, the most probable hypothesis indicated that the Brazilian coastal Parnamirim population (PNM) originated from Florida (VRB). Subsequent admixture events between Florida (VRB) and Parnamirim (PNM) (58% and 42% respectively) gave origin to the Rio de Janeiro state/Jurujuba (JRB) population. Among the Brazilian populations, the Amazonas State sample from Manaus (MAN) appeared to be the most differentiated. Its most likely origin (analysis 3d) was an admixture event between VRB-Florida and JP (52% and 48%, respectively). In Argentina, the mosquito has been recorded in Misiones Province near the border with Brazil. Thus, we tested whether the Misiones sample (MIA) was also a derivation from the Brazilian and/or Florida populations. The Argentinian mosquitoes appear to have originated as a split from the Brazilian JRB population (analysis 3c, Fig. 3c).

#### Central America

The relatively recent demographic histories of the populations Tapachula in Mexico (MXC) and Colòn in Panama (PAN) were considered under the hypothesis of a North American and/or a Brazilian derivation (analyses 4a, b, Fig. 3e). The most likely origin of MXC was a split from Florida-VRB. Regarding PAN, the most statistically supported scenario suggests that it originated from an admixture between VRB in Florida and Brazilian PNM.

#### Congo in Central Africa

The origin of the Brazzaville population in Congo was tested in analysis 5 (Fig. 3f). A very high posterior probability suggests that this central African population split from the Brazilian population of MAN (Manaus).

### Population ancestry affects vector competence for CHIKV

Based on the above results, we selected 13 *Ae. albopictus* populations for which vector competence data obtained from experimental CHIKV infections were available (Table 1, Supplementary Table 4) to explore whether, and to what extent, the degree of ancestry detected among the populations impacted their dissemination efficiency (DE) and transmission efficiency (TE) for CHIKV. DE and TE respectively refer to the proportion of mosquitoes with infectious viral particles in the head and in the saliva among the number of mosquitoes tested.

**Fig. 4:**
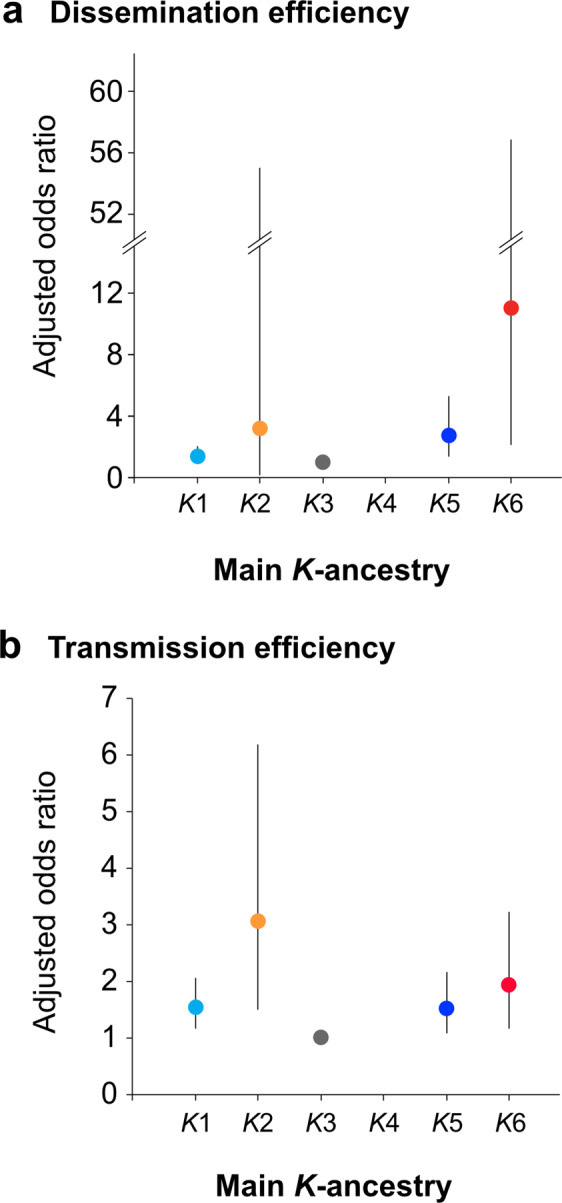
Graphical representation of the influence of *K*-ancestry on dissemination and transmission efficiencies. **a** Dissemination efficiency (*n* = 443, 13, 498, 0, 256 and 80 for *K*1–*K*6, respectively, overall *P* = 0.003); and **b** transmission efficiency (*n* = 401, 52, 424, 0, 203 and 79 for *K*1–*K*6, respectively, overall *P* = 0.0006). Circles represent the adjusted Odds Ratio estimate and the bar its 95% confidence interval obtained from the logistic regression presented in Tables 3 and 4, respectively.

A total of 1290 mosquitoes were considered in the dissemination analysis of which 1120 (86.8%) showed disseminated infection. CHIKV genotypes, dpi and blood titre were significantly associated with DE in univariate analysis (Table 3). When populations were grouped according to their major *K*-ancestry (as listed in Table 2), DE significantly differed according to *K*-ancestry in univariate analysis (*P* = 0.0001, Table 3). This remained true after adjusting on CHIKV genotypes, dpi and blood meal titre (Supplementary Table 5, Fig. 4a). As compared to mosquitoes with main *K*3-ancestry (North and Central America: OR = 1), those with major *K*6 (IT1, Italy), *K*2 (BL-France), *K*5 (Brazilian MAN and CONG1) and *K*1 (South America) ancestries showed significantly higher dissemination efficiencies (Fig. 4a). Dissemination of CHIKV strains from Asian genotype (CHIKV-ASIA) and, to a lesser degree, those of CHIKV ECSA 226 V strains significantly decreased across the *K*-ancestry lineages: from *K*6 (IT1, Italy) to the derived *K*2 (BL, France), to *K*5 (Manaus, Congo) to *K*1 (South America) and *K*3 (North and Central America) which displayed similar efficiencies. Another approach consisted in introducing all 6 *K-*ancestries when dichotomised. In univariate analysis, levels of *K*1, *K*5 and *K*6-ancestries >0.25 were significantly associated with DE. It appeared that these three dichotomised factors defined only four distinct population profiles (low *K*1, *K*5 and *K*6-ancestries, high *K*1-ancestry only, high *K*5-ancestry only and high *K*6-ancestry only); to simplify model interpretation, a new variable was defined allowing comparison of these 4 populations (Table 3). After adjusting for CHIKV genotype, dpi and blood meal titre, as compared to the populations with lower *K*5 and *K*6-ancestries, those with higher *K*5 or *K*6 presented higher DE (Table 3). Both multivariate models showed a tendency for DE to decrease from populations with major *K*6-ancestry to major *K*5-ancestry and finally to the other populations.

**Table 3:** Dissemination efficiency of CHIKV in *Ae. albopictus* lineages (Adjusted logistic regression).

		Model 1		Model 2	
Factor	Variable	Adjusted OR (95% CI)^a^	*P*	Adjusted OR (95% CI)^a^	*P*
Virus genotype^b^	ASIA	3.14 (1.58–6.20)	**<0.001**	3.29 (1.64–6.61)	**<0.001**
	ECSA	1		1	
	ECSA E1-226V	2.52 (1.68–3.80)		2.64 (1.75–3.98)	
Days post-infection	6-7	1	0.47	1	0.54
	10	1.15 (0.79–1.68)		1.13 (0.77–1.65)	
Blood titre	6.5	0.06 (0.02– 0.13)	**<0.001**	0.05 (0.02–0.13)	**<0.001**
	7.5	1		1	
Main *K*-ancestry	1	1.32 (0.87–2.00)	**0.003**	–	–
	2	3.18 (0.18–55.20)		–	
	3	1		–	
	4	–		–	
	5	2.69 (1.36–5.30)		–	
	6	10.96 (2.11–56.85)		–	
*K*-ancestry profile	*K*1, *K*5 and *K*6 ≤ 0.25	–	–	1	**0.002**
	Only *K*1 > 0.25	–		1.29 (0.85–1.95)	
	Only *K*5 > 0.25	–		2.75 (1.38–5.51)	
	Only *K*6 > 0.25	–		15.75 (2.14–116.11)	

^a^*OR* odds ratio, *CI* confidence interval.

^b^ASIA: CHIKV strains from Asian genotype. ECSA: CHIKV strains from East-Central-South African genotype harbouring an alanine at position 226 of E1 glycoprotein. ECSA E1-226V: CHIKV strains from East-Central-South-African genotype harbouring a valine at position 226 of E1 glycoprotein. Bold *P*-values are significant (*P* < 0.05).

Transmission efficiency (TE) for CHIKV was evaluated in 1159 mosquitoes of which 58.0% (672) could transmit the virus. CHIKV genotypes were significantly associated with TE, with ECSA E1-226V strains being more efficient (*P* = 0.0002) and ECSA (E1-226A) at a lower degree, while no association with TE was detected according to dpi and blood meal titres (Supplementary Table 6). Again, the *K*-ancestry lineages displayed different TEs (Supplementary Table 6). In univariate analysis, as compared to populations with major *K*3-ancestry, all other populations presented greater TEs. This remained true after adjusting for CHIKV genotype, as populations with major *K*2-ancestry (La Réunion, PROV and the derived French BL) displayed the greatest TEs (Table 4 and Fig. 4b). As for DE, another approach consisted in looking at the level of each six *K*-ancestry when dichotomised. In univariate analysis, the levels of *K*1, *K*2 and *K*3-ancestries were all significantly associated with TE (Supplementary Table 6). In multivariate analysis, a higher level of *K*3-ancestry was associated with lower TE, whereas higher levels of *K*2 were associated with a greater TE (Table 4). Both multivariate models showed a tendency for TE to reduce from *K*2 to *K*1 and finally *K*3.

**Table 4:** Transmission efficiency in *Ae. albopictus* lineages (Adjusted logistic regression).

		Model 1		Model 2	
Factor	Variable	Adjusted OR (95% CI)^a^	*P*	Adjusted OR (95% CI)^a^	*P*
Virus genotype^b^	ASIA	0.99 (0.68–1.42)	**0.0072**	0.96 (0.66–1.38)	**0.0015**
	ECSA	1		1	
	ECSA E1-226V	1.48 (1.12–1.95)		1.50 (1.14–1.98)	
Main *K*-ancestry	1	1.55 (1.17–2.07)	**0.0006**	–	-
	2	3.06 (1.51–6.19)		–	
	3	1		–	
	4	–		–	
	5	1.53 (1.09–2.16)		–	
	6	1.94 (1.17–3.22)		–	
*K*1 ancestry	≤0.25	–	–	1	0.51
	>0.25	–		1.10 (0.82–1.48)	
*K*2 ancestry	≤0.25	–	–	1	**0.049**
	>0.25	–		2.06 (1.00–4.25)	
*K*3 ancestry	≤0.25	–	–	1	**0.025**
	>0.25	–		0.73 (0.56–0.93)	

^a^*OR* odds ratio, *CI* confidence interval.

^b^ASIA: CHIKV strains from Asian genotype. ECSA: CHIKV strains from East-Central-South African genotype harbouring an alanine at position 226 of E1 glycoprotein. ECSA E1-226V: CHIKV strains from East-Central-South-African genotype harbouring a valine at position 226 of E1 glycoprotein. Bold *P*-values are significant (*P* < 0.05).

Also, on the basis of population profiles, when compared in mosquitoes with low *K*2 and *K*3-ancestries, transmission was significantly higher in those with high *K*2-ancestry and lower in those with higher *K*3-ancestry (Supplementary Table 6).

## Discussion

This study provides a picture of the major events which determined, at the macrogeographic level, the demographic history of the adventive populations established in the Indian Ocean island of La Réunion, in the Mediterranean area, in the Americas and in Central Africa, as a consequence of the rapid west-oriented invasive processes of *Ae. albopictus* out of South East Asia starting from the 18th century. We notably found that (1) admixture events had a major role in shaping the genetic makeup of the globally distributed adventive populations, impacting their demographic histories, and (2) the population demographic history impacted their ability to transmit CHIKV. It follows that knowledge of the relationship between demographic history and vector competence is crucial for assessing the risk of arbovirus outbreaks in areas where this mosquito has become established.

**Fig. 5:**
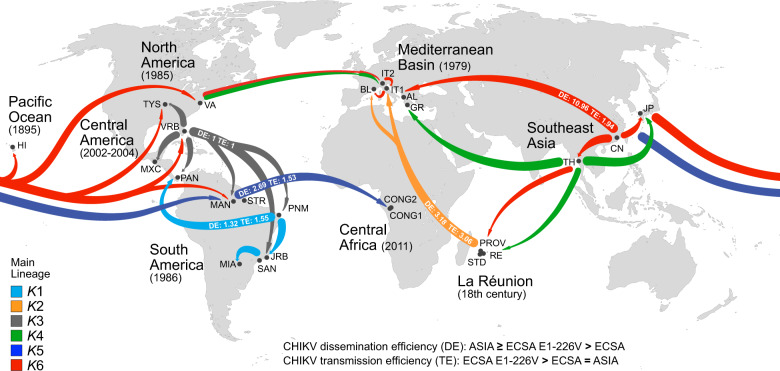
Geographical representation of the global spread of *Ae. albopictus* highlighting the co-ancestry of the different derived populations and the dissemination and transmission efficiencies for Chikungunya virus (CHIKV). ECSA E1-226V: CHIKV strains from East-Central-South African genotype harbouring a valine at position 226 of E1 glycoprotein. ECSA: CHIKV strains from East-Central-South African genotype harbouring an alanine at position 226 of E1 glycoprotein. ASIA: CHIKV strains from Asian genotype.

As far as it concerns the demographic history of this mosquito, six gene pools/lineages (clusters) were detected across the invaded regions at the macrogeographic level. The data clearly suggest that the invasion process of this mosquito fits a demographic model of historical lineage diversification through admixture events among different genetic compositions as summarized in Fig. 5. The lineage divergence times we inferred from genetic data (ABC, STRUCTURE) are congruent with the available historical documentation on the different steps of the invasion process, thus providing a picture of the history of this mosquito outside its home range over the last 200 years. In Southeast Asia, a high level of lineage-sharing is displayed by the ancestral Chinese and, to a lesser degree, by the derived Thai and Japanese populations, supporting high coancestry in this home range area (Fig. 2). The historical trade networks across East Asian maritime space, the ‘China seas’, may have promoted the diffusion of propagules to Thailand and Japan resulting in an increase in diversity and favouring expansion and adaptation. As illustrated in Fig. 5, we inferred that *K*6 (red) and *K*4 (green) were the first ancestral lineages that historically emerged from Southeast Asia. *K*6, highly represented in China, is the lineage that may correspond to the primary invasion route within East Asia (Thailand and Japan) and, out of this area, to Indian Ocean La Réunion in the 18th century, to Hawaii in 1895, to the Mediterranean area starting from 1979 and to the USA in 1985^40^. *K*4 is highly present in Thailand and contributed to the ancient colonization of the west coast of La Réunion, to the USA, and to the recent outbreaks in the Mediterranean area, Greece and partially to Cesena (IT1)^32^. A spatial extension of *K*6 and *K*4 from the western side of La Réunion (St Pierre, RE) to the eastern part of the island (PROV and STD) gave rise to the *K*2 lineage diversification (orange). Habitat structuring and strong climatic differences between the east and western coasts of La Réunion may have supported this diversification^41,42^. *K*2 expanded its range out of La Réunion and became established in the French Mediterranean area of Bar Sur Loup (BL) in 2004. La Réunion is a major trading partner of Continental France and most of this international trade passes through the main port and the international airport at St Denis on the north-east coast where *K*2 was established (PROV and STD). *K*6 and *K*4 were almost lost in the invasion process of the Americas. In North America, they contributed to the diversification of the *K*3 lineage (grey in Fig. 5) which is the major component of the *Ae. albopictus* genomes in Florida and Missouri. Indeed, within a year of its introduction in Texas in 1985^43^, it was found in Florida and then rapidly expanded throughout the south-east coast and part of north-central and north-east USA due to internal trade and multiple introductions from Japan/South Asia and Hawaii. According to our data and historical records, Florida played a key role in introducing this mosquito in South America in 1986 and later, in Central America, Mexico and Panama in 2002 and 2004, respectively^44,45^. Thus, Florida can be considered as a successful bridgehead invasive population which favoured the extension of *K*3 (grey in Fig. 5) in Central America and the divergence of *K*1 (cyan) in Brazil with its subsequent diffusion within that country and in Argentina. In South America, the Rio Grande do Norte (PNM) and Rio de Janeiro (JRB) states appear to have played a role in the spread of the *K*1 lineage. A particular case is provided by the genetic signature of Brazilian mosquitoes present in the Central Amazonian state (Manaus) since 2002. Unlike the other Brazilian mosquitoes, they display a higher proportion of the ancient Southeast Asian *K*5 lineage (0.450), with respect to the *K*1 Brazilian component which is unexpectedly low (0.240) and that of North Central America, *K*3, is even lower (0.167). The high *K*5 lineage component may be explained by the contribution that Southeast Asia/Japan provided to the origin of Manaus mosquitoes^46^. Manaus is one of the four free trade areas in Brazil and its port is a commercial centre for ocean-going shipping and the main transport hub for the Amazon basin. We found the *K*5 lineage well established in mosquitoes from Congo in Central Africa as a derivation from Amazonian Manaus. How this area could be implicated in the introduction of this mosquito to Congo is an open question although it is noteworthy that this inference was derived from a sample collected in 2011; the year of the first record of this mosquito in this area. Several authors have also proposed that propagules originating from South America could have contributed to an expansion in Africa.

Finally, as summarised in Fig. 5, the old colonized Mediterranean area appears to be a mixing pot where the introduction of different lineages or their components resulted in the mixing of different genomes with the consequent heterogeneity observed among the populations.

Geographic variation in vector competence for different CHIKV genotypes has been observed among *Ae. albopictus* populations, highlighting that distinct barriers to dissemination and to transmission may operate even at small geographic scales depending on the interactions between the vector’s genetic background and the viral genotype^15,37,39,47^. Our data demonstrated that the ability of an *Ae. albopictus* population to disseminate or transmit the different CHIKV genotypes is strongly associated with their degree of ancestry within a genetic lineage. It follows that if populations from different geographical areas are related by their demographic history notably in terms of ancestry, they will display similar levels of vector competence for a given CHIKV strain. This is true for the La Réunion PROV, STD and the French Mediterranean BL populations in *K*2; the Brazilian Amazonian Manaus and Congo populations in *K*5, and the North and Central American populations in *K*3 (Tables 3 and 4). On the other hand, this phenomenon generates variation in competence within regions in which different lineages or admixture of lineages coexist, like in the Mediterranean area.

If we consider the historical dynamics of *Ae. albopictus* expansion and the temporal and spatial history of CHIKV evolution and spread, it appears that they are bound by convergent adaptive evolution^39,48,49^. Indeed, the historical sequential divergence and admixture of *Ae. albopictus* lineages out of Southeast Asia determined changes in the level of populations’ competence for CHIKV, thus facilitating the establishment of CHIKV transmission cycles in new regions. All the mosquitoes from the genetic lineages examined were able to disseminate the Asian and ECSA E1-226V CHIKV genotypes, better than ECSA (E1-226A). However, there is a progressive and drastic loss of permissiveness of the mosquito barriers for CHIKV dissemination (i.e. antiviral immune responses at the midgut epithelium) regarding the Asian and ECSA E1-226V genotypes that parallels the historical lineage divergence from the ancestral Asian lineage *K*6 to *K*2, to *K*5 and finally, to the American *K*1 and *K*3 lineages. Indeed, the higher abilities to disseminate CHIKV Asian and ECSA E1-226V genotypes were observed in populations with higher levels of the ancestral *K*6 and *K*5 lineages (Fig. 5), suggesting that the loss or modification of ancestral components in the genome of invading populations resulted in enhanced tissue barriers to CHIKV dissemination in mosquitoes. In contrast to their dissemination efficiency, the lineages displayed reduced transmission efficiency for CHIKV-ASIA genotype with respect to both ECSA genotypes, especially ECSA E1-226V. These results can be explained by the constrained ability of Asian CHIKV strains to adapt to *Ae. albopictus* due to negative epistatic interactions between the E1 residue 98 (that harbours a threonine in Asian strains; E1-98T) and the E1-226 position^49^. The highest transmission efficiency was displayed by La Réunion *K*2 lineage followed by *K*6 which retains around two-thirds of transmission efficiency. Interestingly, the *K*2 lineage differentiated in La Réunion where the CHIKV E1-226V genotype was identified for the first time in 2005 and established as an adaptive mutation of ECSA lineage for this mosquito^50,51^. ECSA E1-226V, with a valine instead of an alanine at position 226 A of the E1 glycoprotein, significantly increased viral replication in midgut infection and transmission. The association of this mutation with *Ae. albopictus* invasiveness impacted CHIKV diffusion in a sort of convergent adaptive evolution between vector populations and viral genomes^49^. Moreover, the ECSA E1-226V mutation is thought to have arisen independently in other areas where *Ae. albopictus* is present, supporting this convergent evolution hypothesis^49,52^. We have found that the higher the level of the *K*2 lineage in a population is, the higher is its ability to transmit the ECSA E1-226V genotype. After 2007, the ECSA E1-226V genotype was identified in Southeast Asia and quickly spread, supported by the transmission efficiency of the *Ae. albopictus* ancestral lineages (*K*6 and *K*5), inducing major chikungunya epidemics^53^. Prior to 2007, *Ae. albopictus* had never been incriminated in outbreaks involving Asian CHIKV strains, despite the massive presence of the mosquito in this region. These outbreaks were vectored by *Ae. aegypti* which is also abundant in the area. One plausible reason would be that, as explained above, Asian strains of CHIKV were constrained in their ability to adapt to *Ae. albopictus* via acquisition of the E1-A226V mutation^49^. This inability could have facilitated the establishment of the *Ae. albopictus*-adapted CHIKV strains that harbour the E1-226V substitution in the region, leading to the ongoing shift from Asian to ECSA E1-A226V genotypes in Southeast Asia^49^.

In the Mediterranean area, no CHIKV Asian genotype has been related to autochthonous transmission despite the intense influx of travellers returning from Southeast Asia and the Americas. It has been shown that exposure of European *Ae. albopictus* to low temperatures (20 °C) significantly increased the extrinsic incubation period and reduced the transmission efficiency of Asian CHIKV strains, while for strains from ECSA lineages these parameters remained unaffected^15,54^. Although other factors influencing vectorial capacity may interact and determine that an infectious bite is received by a susceptible human host^7^, these results firstly highlight the importance of three-way interactions between temperature, the mosquito population and the viral genotype. Secondly, they suggest a higher risk for ECSA strains to emerge in Europe. In addition, the presence in this old-colonized area of different *Ae. albopictus* lineages that are highly competent for ECSA genotypes (Supplementary Table 5; Supplementary Table 6), coupled to a growing number of imported cases^55^ led to autochthonous chikungunya transmission in France^56–58^ and Italy in 2007 and 2017^59–62^. In all these autochthonous transmissions, the strains involved belonged exclusively to the ECSA lineage, which supports the CHIKV emergence hypothesis enounced above. Nevertheless, the heterogeneity of *Ae. albopictus* genomes in the Mediterranean region has resulted in a patchwork of populations displaying different levels of competence which makes risk assessment for CHIKV highly unpredictable at a finer scale in the area^63^. Beyond vector competence, context-specific differences in parameters with greater impact on vectorial capacity (i.e. longevity, density, host feeding preference)^7^ may challenge risk prediction for arboviruses and should be taken into account in further studies.

The most recent divergent American lineages, *K*1 (South America) and especially, the North and Central American lineage *K*3, displayed the lowest CHIKV dissemination and transmission efficiencies of all studied populations. Indeed, *K*3 is a heterogeneous lineage with USA populations such as Florida/Vero Beach that displayed lower CHIKV dissemination and transmission efficiencies (Supplementary Table 5; Supplementary Table 6). Vega-Rúa and colleagues^37^ and Honório and colleagues^47^ using the three CHIKV strains of this study (ASIA, ECSA E1-226A and ECSA E1-226V) performed comparative analyses of vector competence using *Ae. albopictus* and *Ae. aegypti* populations across the Americas. All the populations of both species were susceptible to infection and dissemination of the different CHIKV genotypes. However, the transmission rates were heterogeneous even among populations from the same species with *Ae. albopictus* from Brazil, and generally from South America, having higher transmission efficiency for ECSA genotypes when compared to their counterparts from Florida, USA. The first detection of CHIKV in the Americas was recorded in the Caribbean in late 2013, followed by a dramatic spread of the virus throughout the continent. It has been stated that CHIKV arrived in Brazil through two independent introductions: the Asian genotype from the Caribbean entered through the northern region, while the African ECSA genotype was imported through the north-east region^64^. Following their initial introduction, both genotypes established and expanded their range with CHIKV-ASIA being the prevalent genotype causing several outbreaks in relation to the presence of *Ae. aegypti* that was the main vector implicated in the transmission^15,37^. In 2014, local transmission of CHIKV-ASIA was detected in the Amazon region. A distinct lineage CHIKV-ECSA was detected simultaneously and it rapidly spread inducing replacement of the Asian genotype in the Brazilian Amazonian region^65^. Whether this replacement was due to the spread of *Ae. albopictus* in this region and its transmission capacity for these particular ECSA strains, which do not harbour the E1-226V mutation, is an open question that deserves further investigation.

The ancestral *K*5 lineage is differentiated in the African populations from Congo that became established in 2011 as a derivation of the *K*5 Amazonian component. Vazeille and colleagues in 2016^38^ demonstrated that *Ae. albopictus* newly introduced in Congo were unable to transmit the ECSA E1-226V genotype with the same efficiency as La Réunion mosquitoes, highlighting that genetic differentiation among these two populations may have impacted their vector competence. Our results are in agreement with this hypothesis: Congo and La Réunion belong to two well differentiated lineages *K*5 and *K*2, respectively, with Congo having half the ECSA E1-226V transmission efficiency when compared to the La Réunion *K*2 lineage.

In conclusion, it is clear from these data that the demographic history of *Ae. albopictus* populations is tightly related to CHIKV genotypes in a sort of adaptive coevolution, where genotype-by-genotype interactions influenced the corresponding *Ae. albopictus* vector competence phenotypes for this virus. It follows that the knowledge of the vector demographic history dynamics combined with vector competence data can provide an accurate risk map for CHIKV. However, in addition to these genetic interactions, we must consider that the spread of this mosquito occurred across different environmental conditions. Thus, the variation in competence between the eco-geographic populations may be impacted by the interactions between unexplored genomic factors^66,67^ within genomes and lineages, and complex environmental landscapes.

## Methods

### Mosquito collections

Twenty-five mosquito populations were considered. Three were from the ancestral East Asian area and 22 were sampled across much of the expansion area: La Réunion in the Indian Ocean, the Mediterranean basin, Hawaii in the Pacific Ocean, North, Central and South America, and Central Africa (Table 1). The samples from Southeast Asia (Japan, China, Thailand), La Réunion (St Pierre), the Mediterranean basin (Albania, Greece, Italy1, Italy2), Hawaii and North America (Virginia) were previously described by our laboratory in terms of microsatellite allele frequencies, genetic structure and demographic history^32^. Their integration in this analysis aimed at obtaining a deeper genetic portrait of the demographic history of this species during its invasion process. For the 25 collection sites, samples were collected as eggs at the peak of density to avoid any seasonality effects due to the presence of diapause or pre-diapause periods^10^. As described in Manni and colleagues^32^, a standardized sampling protocol was adopted to minimize inbreeding effects at each breeding site. In each locality, 15–17 ovitraps were placed at a distance of least 500 m one to another, and at least 40 eggs/ovitrap were obtained.

Adults from each field collection were identified using morphological keys^68^. The mosquito egg collections in the Americas, La Réunion, France, the North of Italy and in Congo/Central Africa were separated in two batches: one batch was used for population genetic studies and the remaining batch, that gave rise to several hundred individuals, was amplified in the laboratory under controlled conditions to obtain the offspring that was used in vector competence assessments for CHIKV (Table 1). Thus, the microsatellite genotyping analyses were based on an average of 30 G0 individuals for each population sample, while vector competence assays were performed on the corresponding G1 mosquito populations, with the exception of Brazaville (G2) and Cesena (G3).

### Microsatellite analyses

We used previously validated SSRs as highly polymorphic markers to provide continuity with our published *Ae. albopictus* population data and because their use allowed accurate biogeographic data analyses especially at macrogeographic scale. Indeed, the availability of a population SSR genotype dataset was a reliable resource for ABC simulations as SSRs represented a good balance between being well characterized (i.e. in terms of mutation model) and having sufficient genetic diversity to provide statistical power, as we previously assessed^31^.

We used 11 highly informative SSR loci applied to 702 individuals from 25 localities to produce a SSR genotyping dataset with no major bias in statistical analyses^69^.

On this basis, genomic DNA extracted from each mosquito^70^ was genotyped at the following SSR loci: Aealbmic1, 2, 3, 5, 6, 9, 11, 14, 15, 16 and 17^31^. These loci were chosen due to their high polymorphism and because they are spread across the genome (they map to different scaffolds of the *Ae. albopictus* Foshan genome (ref. ^71^ and our unpublished data)). They have proven to be efficient markers to detect variability, even in relatively small samples^32^. PCR amplifications and fragment identifications were performed as previously described^31^. In order to account for genotyping errors, automated binning of allele lengths was performed with TANDEM v1.09^72^ followed by manual checking. When microsatellite amplification was not successful, or allele scoring was unclear, a new DNA extraction was performed. The generated SSR genotype dataset, in addition to offering continuity with previous studies, allows the formulation of reliable models of recent evolutionary history because of their well‐documented mutation rates^73^, high molecular diversity and high minor allele frequencies.

### Variability and genetic diversity analyses

Linkage disequilibrium between pairs of loci in each sample (100 batches, 1000 iterations per batch) and deviation from within-population Hardy–Weinberg equilibrium (HWE) at each locus/sample combination were tested with GENEPOP V. 4.2^74^. The statistical significance was assessed following Bonferroni corrections^75^. Variation within each locality was estimated in terms of average number of alleles (na), private alleles (np) and their respective frequencies (Ap) using GenAlEx 6.5^76^. The average number of alleles (na/n) and private alleles (np/n) were also computed at the individual level. Gene diversity (Hs) and allelic richness (Rs) were computed with the program FSTAT V.2.9.3.2^77^. Observed (Ho) and unbiased expected heterozygosity (uHe) values and Pairwise-*F*_ST_ were computed using Microsatellite Analyzer (MSA) V.4.05^78^. The statistical significance of each *F*_ST_ value was assessed by comparison of the assessed value with the value obtained using 10,000 matrix permutations and Bonferroni corrections.

### Population structure

The structure and degree of ancestry in the 25 population samples were inferred using the Bayesian clustering analysis implemented in STRUCTURE V 2.3.2 under the admixture model and assuming independent allele frequencies. The “burn-in” phase was set to 500,000 iterations, then 1,000,000 Markov Chain Monte Carlo (MCMC) replications were considered. For each number of possible clusters (*K*), 20 independent runs were repeated. The cluster (*K*) interval was set between 1 and 25 (i.e. the number of considered populations).

The results were analysed using STRUCTURE HARVESTER^79^ and the most likely number of clusters was determined by plotting the log probability (L(*K*))^80^, as well as the Δ*K* over all the runs^81^. Once the best number of clusters, *K*, was inferred, CLUMPP software^82^ was used to merge the 20 runs and the results were plotted in DISTRUCT^83^.

### Analyses of demographic History

The Approximate Bayesian Computation (ABC) method, as implemented in the software DIYABC v2.1, was used to disentangle the complex invasion patterns of *Ae. albopictus* at the macro-geographic level. This approach allows comparisons between competing hypotheses regarding the divergence of the populations on a global scale. DIYABC v2.1 implements a standard ABC method (ABC-LDA) which, although being more expensive computationally, was shown to perform comparably (for posterior probability) or slightly better (for prior error rate) than the more recent random forest method (ABC-RF)^84^ when large reference tables are used^85^, as in our case. Because of the huge number of possible evolutionary scenarios (25 factorial, i.e. 1.55e^25^) that could be tested with the 25 populations considered, we adopted a step-by-step approach considering subgroups of populations, starting from the ancestral Southeast Asian region to the invaded areas. Based on our published data, we considered the previously characterized samples from China, Thailand and Japan as representative populations from countries within the Southeast Asian native range.

Several competing invasion scenarios were considered taking into account the available historical information regarding the first records in invaded countries and population genetic data. The posterior probability for each scenario was computed. The competing scenarios were set using prior definitions and distribution of demographic parameters, as described in Supplementary Table 7. For some of these analyses, we referred to and reran the scenarios that we had previously validated^32^. We took into account the effective population size, the time range in which split or admixure events occurred, the number of founders that contributed to the establishment of adventive populations, the duration of the eventual bottlenecks occurring during colonization and the rate of admixture, if considered^32^. When no prior information was available, the prior parameters were kept deliberately broad. For the effective population sizes, broad priors [500–100,000] with uniform distributions were chosen for all the populations. For each event, the time range chosen was less broad if it was supported by historical data. The timing of events was expressed in numbers of generations back in time. Considering that the number of generations/year is dependent on bioclimatic conditions, we assumed around 4–7 generations/year in temperate regions, and 12–17 generations/year in tropical regions. In the cases where the climate situation is not so clear-cut, the broadest interval was considered (i.e. 4–17 generations/year).

As mentioned before^32^, although *Ae. albopictus* has great potential for rapid population growth^86^, uncertainty regarding the duration of the bottleneck was taken into account. Therefore, we assumed a bottleneck period with a uniform prior distribution bounded between 0 and 50 generations. During colonization, the number of founder individuals for each colonization event was described as *NF* and its size was drawn from a uniform distribution bounded between 1 and 100 individuals. If necessary, the rate of admixture between two populations was drawn in a uniform distribution and set from 0.001 to 0.999. For the genetic parameters, the microsatellite Generalized Stepwise Mutation model was considered, and a mean microsatellite mutation rate across loci was set between 10^−5^ and 10^−3^. The parameter of geometric distribution was specified with a uniform prior distribution bounded between 0.1–0.3. For each locus, the above considered mutation parameters were allowed to vary using Gamma distributions. Finally, the possibility of single nucleotide insertion/deletion mutations were considered with a mean frequency of 10^−8^–10^−5^ (Supplementary Table 7)^32^.

The genetic variation within and among the populations was summarized using sets of statistics conventionally used in ABC analyses: the mean number of alleles per locus, the mean expected heterozygosity, the mean allelic size variance, the overall pairwise *F*_ST_ values, and the Garza-Williamson index (number of alleles in a population divided by the range in allele size)^87^. This index helps to discriminate populations that have experienced recent losses in genetic variability from those that have been stable for a long time. One million simulated datasets were generated for each scenario. Competing scenarios were compared by calculating their posterior probabilities using a polychotomous logistic regression on the 1% of simulated data closest to the observed data^88^. Confidence in scenario choice was evaluated by computing type I and type II errors^89^. Once the most likely scenario was identified for each analysis, the posterior distributions of genetic and demographic parameters were estimated. This was achieved by computing a local linear regression on the 1% of the simulated data closest to our observed dataset, after applying a logit transformation to the parameters value. The goodness-of-fit of the estimation procedure was also evaluated by performing a model checking computation by generating 1000 pseudo-observed data sets with parameter values drawn from the posterior distribution given the most likely scenario^32^.

### Vector competence data for CHIKV strains

Vector competence for CHIKV was assessed on adults derived from field-collected eggs from the Americas, La Réunion Island, France, North Italy and Congo as described above (Table 1). Batches of 5–7-day-old females were mono-infected using an artificial feeding system (Hemotek Ltd®, Blackburn, UK) with low passaged CHIKV strains (2–3 passages) belonging to either East-Central-South African genotype (ECSA) or the Asian genotype (see section Viral Strains for more details). Fully engorged females were kept for further estimation of DE, TE and their saliva was collected at the selected days post-infection (Supplementary Table 4) as described by Vega-Rúa and collegues^37^. The infectious status of head and saliva samples was determined by titration assays^90^. The blood meal titres, viral strains, days post-infection (dpi) as well as DE and TE data are summarized in Supplementary Table 4.

### Viral strains

Six CHIKV strains isolated from human cases were used in infection assays. Five strains belonged to the ECSA genotype: CHIKV_0621 (Accession number AM258992) and CHIKV_05115 (Accession number AM258990) isolated in La Réunion, CHIKV_1909 (Accession number FR846305) from Southeast France, CHIKV_DRC and CHIKV_Congo from Congo^38^. All these ECSA strains were split into two different genotype groups according to the amino acid substitution at 226 position of E1 envelope protein: the group named ‘ECSA’ that contains the substitution E1-226A (Alanine) and the group named ‘ECSA E1-226V’ that contains a Valine at the same position. This mutation allows an increased transmission by more than 50-fold in *Ae. albopictus*^50,51^. The strain from Asian genotype used, CHIKV_NC, was isolated in New Caledonia in 2011.

### Association between population ancestry and CHIKV competence

The effect of viral genotype, dpi, and population ancestry on DE and TE were investigated using logistic regression models. In case of complete prediction of the outcome in one category, a penalized likelihood estimation was considered^91^. For TE, only mosquitoes that had disseminated CHIKV infections were considered. The Odds Ratio (OR) has been considered as a measure of the risk of CHIKV dissemination and transmission. The main factor of interest was the degree of ancestry among populations (*K*-ancestry) and two approaches were used. First, each geographical population is defined by its major *K*-ancestry (Table 2). Second, all 6 *K*-ancestry components were considered but dichotomised as above or below the threshold of 0.25; this threshold was deliberately chosen so that some populations could be characterized by a mixture of several *K*-ancestors. For both dissemination and transmission, factors that presented *P*-values < 0.05 in univariate analysis were introduced in multivariate models. Two multivariate models were considered using either the major *K*-ancestry or the binary *K*-ancestry factors.

### Statistics and reproducibility

The details regarding the statistical analyses, the tests, software used, sample sizes and number of replicates are described in the relevant sections of the “Methods”.

### Reporting summary

Further information on research design is available in the Nature Research Reporting Summary linked to this article.

## Supplementary information

Supplementary Information

Supplementary Data 1

Description of Additional Supplementary Files

Reporting Summary

Peer Review File

## Data Availability

The datasets generated during and analysed during the current study are available in the Open Science Framework repository: The raw genotype data has been submitted to the Open Science Framework data repository: https://osf.io/g5pt3/?view_only=ee95053bf7cf496292320e11df0476a8.

## Code availability

Stata code for the logistic regressions will be made available on request.

## References

[1] Kraemer MU (2015). The global distribution of the arbovirus vectors *Aedes aegypti* and *Ae. albopictus*. Elife.

[2] Mogi M, Armbruster P, Tuno N, Campos R, Eritja R (2015). Simple indices provide insight to climate attributes delineating the geographic range of *Aedes albopictus* (Diptera: Culicidae) prior to worldwide invasion. J. Med. Entomol..

[3] Delatte H, Gimonneau G, Triboire A, Fontenille D (2009). Influence of temperature on immature development, survival, longevity, fecundity, and gonotrophic cycles of *Aedes albopictus*, vector of chikungunya and dengue in the Indian Ocean. J. Med. Entomol..

[4] Medlock JM (2012). A review of the invasive mosquitoes in Europe: ecology, public health risks, and control options. Vector Borne Zoonotic Dis..

[5] Brady OJ (2014). Global temperature constraints on *Aedes aegypti* and *Ae. albopictus* persistence and competence for dengue virus transmission. Parasit. Vectors.

[6] Schmidt CA, Comeau G, Monaghan AJ, Williamson DJ, Ernst KC (2018). Effects of desiccation stress on adult female longevity in *Aedes aegypti* and *Ae. albopictus* (Diptera: Culicidae): results of a systematic review and pooled survival analysis. Parasit. Vectors.

[7] Lounibos LP, Kramer LD (2016). Invasiveness of *Aedes aegypti* and *Aedes albopictus* and vectorial capacity for chikungunya virus. J. Infect. Dis..

[8] Poelchau MF, Reynolds JA, Elsik CG, Denlinger DL, Armbruster PA (2013). Deep sequencing reveals complex mechanisms of diapause preparation in the invasive mosquito, *Aedes albopictus*. Proc. R. Soc. B: Biol. Sci..

[9] Urbanski J (2012). Rapid adaptive evolution of photoperiodic response during invasion and range expansion across a climatic gradient. Am. Naturalist.

[10] Lacour G, Chanaud L, L’Ambert G, Hance T (2015). Seasonal synchronization of diapause phases in *Aedes albopictus* (Diptera: Culicidae). PLoS ONE.

[11] Juliano SA, Lounibos LP (2005). Ecology of invasive mosquitoes: effects on resident species and on human health. Ecol. Lett..

[12] Invasive Species Specialist Group (2020) Species profile: *Aedes albopictus*. Downloaded from http://www.iucngisd.org/gisd/species.php?sc=109 on 15-06-2020.

[13] Paupy C, Delatte H, Bagny L, Corbel V, Fontenille D (2009). *Aedes albopictus*, an arbovirus vector: from the darkness to the light. Microbes Infect..

[14] Gasperi G (2012). A new threat looming over the Mediterranean basin: emergence of viral diseases transmitted by *Aedes albopictus* mosquitoes. PLoS Negl. Trop. Dis..

[15] Vega-Rua A (2015). Chikungunya virus transmission potential by local Aedes mosquitoes in the Americas and Europe. PLoS Negl. Trop. Dis..

[16] Wong PSJ, Li MZI, Chong CS, Ng LC, Tan CH (2013). *Aedes* (*Stegomyia*) *albopictus* (Skuse): a potential vector of Zika virus in Singapore. PLos Neg. Trop. Dis..

[17] Gratz NG (2004). Critical review of the vector status of *Aedes albopictus*. Med Vet. Entomol..

[18] Chouin-Carneiro T (2016). Differential susceptibilities of *Aedes aegypti* and *Aedes albopictus* from the Americas to Zika virus. PLoS Negl. Trop. Dis..

[19] Grard G (2014). Zika virus in Gabon (Central Africa)-2007: a new threat from *Aedes albopictus*?. PLoS Negl.Trop. Dis..

[20] Jupille H, Seixas G, Mousson L, Sousa CA, Failloux AB (2016). Zika virus, a new threat for Europe?. PLoS Negl. Trop. Dis..

[21] Armstrong, P. M. et al. Successive blood meals enhance virus dissemination within mosquitoes and increase transmission potential. *Nat. Microbiol.*10.1038/s41564-019-0619-y (2019). 10.1038/s41564-019-0619-y PMC7199921 31819213

[22] Kraemer MUG (2019). Past and future spread of the arbovirus vectors *Aedes aegypti* and *Aedes albopictus*. Nat. Microbiol.

[23] Cunze S, Kochmann J, Koch LK, Klimpel S (2018). Niche conservatism of *Aedes albopictus* and *Aedes aegypti* - two mosquito species with different invasion histories. Sci. Rep..

[24] Zeller H, Van Bortel W, Sudre B (2016). Chikungunya: its History in Africa and Asia and its spread to new regions in 2013–2014. J. Infect. Dis..

[25] Gould EA, Gallian P, De Lamballerie X, Charrel RN (2010). First cases of autochthonous dengue fever and chikungunya fever in France: from bad dream to reality!. Clin. Microbiol. Infect..

[26] Gjenero-Margan I (2011). Autochthonous dengue fever in Croatia, August-September 2010. Eur. Surveill..

[27] Martinet, J. P., Ferté, H., Failloux, A. B., Schaffner, F. & Depaquit, J. Mosquitoes of North-Western Europe as potential vectors of arboviruses: a review. *Viruses*, 10.3390/v11111059 (2019). 10.3390/v11111059 PMC6893686 31739553

[28] Robinson MC (1955). An epidemic of virus disease in Southern Province, Tanganyika Territory, in 1952-53. I. Clinical features. Trans. R. Soc. Trop. Med Hyg..

[29] Volk SM (2010). Genome-scale phylogenetic analyses of chikungunya virus reveal independent emergences of recent epidemics and various evolutionary rates. J. Virol..

[30] Goubert C, Minard G, Vieira C, Boulesteix M (2016). Population genetics of the Asian tiger mosquito *Aedes albopictus*, an invasive vector of human diseases. Heredity (Edinb.).

[31] Manni M (2015). Molecular markers for analyses of intraspecific genetic diversity in the Asian Tiger mosquito, *Aedes albopictus*. Parasit. Vectors.

[32] Manni M (2017). Genetic evidence for a worldwide chaotic dispersion pattern of the arbovirus vector, *Aedes albopictus*. PLoS Negl. Trop. Dis..

[33] Sherpa, S., Blum, M. G. B. & Després, L. Cold adaptation in the Asian tiger mosquito’s native range precedes its invasion success in temperate regions. *Evolution*, 10.1111/evo.13801 (2019). 10.1111/evo.13801 31313825

[34] Sherpa S (2019). Unravelling the invasion history of the Asian tiger mosquito in Europe. Mol. Ecol..

[35] Kotsakiozi P (2017). Population genomics of the Asian tiger mosquito, *Aedes albopictus*: insights into the recent worldwide invasion. Ecol. Evol..

[36] Pichler V (2019). Complex interplay of evolutionary forces shaping population genomic structure of invasive *Aedes albopictus* in southern Europe. PLoS Negl. Trop. Dis..

[37] Vega-Rúa A, Zouache K, Girod R, Failloux AB, Lourenço-de-Oliveira R (2014). High level of vector competence of *Aedes aegypti* and *Aedes albopictus* from ten American countries as a crucial factor in the spread of Chikungunya virus. J. Virol..

[38] Vazeille M (2016). Importance of mosquito “quasispecies” in selecting an epidemic arthropod-borne virus. Sci. Rep..

[39] Zouache K, Failloux AB (2015). Insect-pathogen interactions: contribution of viral adaptation to the emergence of vector-borne diseases, the example of chikungunya. Curr. Opin. Insect Sci..

[40] Hawley WA, Reiter P, Copeland RS, Pumpuni CB, Craig GB (1987). *Aedes albopictus* in North America: probable introduction in used tires from northern Asia. Science.

[41] Paupy C, Girod R, Salvan M, Rodhain F, Failloux AB (2001). Population structure of *Aedes albopictus* from La Réunion Island (Indian Ocean) with respect to susceptibility to a dengue virus. Heredity (Edinb.).

[42] Delatte H (2013). Evidence of habitat structuring *Aedes albopictus* populations in Réunion Island. PLoS Negl. Trop. Dis..

[43] Sprenger D, Wuithiranyagool T (1986). The discovery and distribution of *Aedes albopictus* in Harris County, Texas. J. Am. Mosq. Control Assoc..

[44] Casas-Martínez M, Torres-Estrada JL (2003). First evidence of *Aedes albopictus* (Skuse) in southern Chiapas, Mexico. Emerg. Infect. Dis..

[45] Eskildsen GA (2018). Maternal invasion history of *Aedes aegypti* and *Aedes albopictus* into the Isthmus of Panama: implications for the control of emergent viral disease agents. PLoS ONE.

[46] Maia RT, Scarpassa VM, Maciel-Litaiff LH, Tadei WP (2009). Reduced levels of genetic variation in *Aedes albopictus* (Diptera: Culicidae) from Manaus, Amazonas State, Brazil, based on analysis of the mitochondrial DNA ND5 gene. Genet Mol. Res..

[47] Honório NA, Wiggins K, Câmara DCP, Eastmond B, Alto BW (2018). Chikungunya virus vector competency of Brazilian and Florida mosquito vectors. PLoS Negl. Trop. Dis..

[48] Glushakova LG (2019). Multiplexed kit based on Luminex technology and achievements in synthetic biology discriminates Zika, chikungunya, and dengue viruses in mosquitoes. BMC Infect. Dis..

[49] Tsetsarkin KA (2011). Chikungunya virus emergence is constrained in Asia by lineage-specific adaptive landscapes. Proc. Natl Acad. Sci. USA.

[50] Vazeille M (2007). Two Chikungunya isolates from the outbreak of La Reunion (Indian Ocean) exhibit different patterns of infection in the mosquito, *Aedes albopictus*. PLoS ONE.

[51] Tsetsarkin KA, Vanlandingham DL, McGee CE, Higgs S (2007). A single mutation in chikungunya virus affects vector specificity and epidemic potential. PLoS Pathog..

[52] Coffey LL, Forrester N, Tsetsarkin K, Vasilakis N, Weaver SC (2013). Factors shaping the adaptive landscape for arboviruses: implications for the emergence of disease. Future Microbiol.

[53] Theamboonlers A, Rianthavorn P, Praianantathavorn K, Wuttirattanakowit N, Poovorawan Y (2009). Clinical and molecular characterization of chikungunya virus in South Thailand. Jpn J. Infect. Dis..

[54] Zouache, K. et al. Three-way interactions between mosquito population, viral strain and temperature underlying chikungunya virus transmission potential. *Proc. Biol. Sci.*10.1098/rspb.2014.1078 (2014). 10.1098/rspb.2014.1078 PMC4150320 25122228

[55] Enserink M (2007). Infectious diseases. chikungunya: no longer a third world disease. Science.

[56] Grandadam M (2011). Chikungunya virus, southeastern France. Emerg. Infect. Dis..

[57] Delisle, E. et al. Chikungunya outbreak in Montpellier, France, September to October 2014. *Euro Surveill* **20**, 21108 (2015). 10.2807/1560-7917.es2015.20.17.21108 25955774

[58] Calba, C. et al. Preliminary report of an autochthonous chikungunya outbreak in France, July to September 2017. *Euro Surveill*. **22**, 10.2807/1560-7917.ES.2017.22.39.17-00647 (2017). 10.2807/1560-7917.ES.2017.22.39.17-00647 PMC5709952 29019313

[59] Angelini, R. et al. An outbreak of chikungunya fever in the province of Ravenna, Italy. *Euro Surveill* **12**, E070906.070901, 10.2807/esw.12.36.03260-en (2007). 10.2807/esw.12.36.03260-en 17900424

[60] Rezza G (2007). Infection with chikungunya virus in Italy: an outbreak in a temperate region. Lancet.

[61] Rezza, G. Chikungunya is back in Italy: 2007-2017. *J. Travel Med.*10.1093/jtm/tay004 (2018). 10.1093/jtm/tay004 29669058

[62] Fortuna, C. et al. Vector competence of *Aedes albopictus* for the Indian Ocean lineage (IOL) chikungunya viruses of the 2007 and 2017 outbreaks in Italy: a comparison between strains with and without the E1:A226V mutation. *Euro Surveill.*10.2807/1560-7917.ES.2018.23.22.1800246 (2018). 10.2807/1560-7917.ES.2018.23.22.1800246 PMC6152176 29871722

[63] Mariconti M (2019). Estimating the risk of arbovirus transmission in Southern Europe using vector competence data. Sci. Rep..

[64] Machado LC (2019). Genome sequencing reveals coinfection by multiple chikungunya virus genotypes in a recent outbreak in Brazil. PLoS Negl. Trop. Dis..

[65] Naveca FG (2019). Genomic, epidemiological and digital surveillance of Chikungunya virus in the Brazilian Amazon. PLoS Negl. Trop. Dis..

[66] Ketkar, H., Herman, D. & Wang, P. Genetic determinants of the re-emergence of arboviral diseases. *Viruses*, 10.3390/v11020150 (2019). 10.3390/v11020150 PMC6410223 30759739

[67] Caragata EP, Tikhe CV, Dimopoulos G (2019). Curious entanglements: interactions between mosquitoes, their microbiota, and arboviruses. Curr. Opin. Virol..

[68] Rueda L (2004). Pictorial keys for the identification of mosquitoes (Diptera: Culicidae) associated with dengue virus transmission. Zootaxa.

[69] Guichoux E (2011). Current trends in microsatellite genotyping. Mol. Ecol. Resour..

[70] Baruffi L (1995). Polymorphism within and between populations of Ceratitis capitata: comparison between RAPD and multilocus enzyme electrophoresis data. Heredity (Edinb.).

[71] Chen XG (2015). Genome sequence of the Asian Tiger mosquito, *Aedes albopictus*, reveals insights into its biology, genetics, and evolution. Proc. Natl Acad. Sci. USA.

[72] Matschiner M, Salzburger W (2009). TANDEM: integrating automated allele binning into genetics and genomics workflows. Bioinformatics.

[73] Buschiazzo E, Gemmell NJ (2006). The rise, fall and renaissance of microsatellites in eukaryotic genomes. Bioessays.

[74] Raymond M, Rousset F (1995). Genepop (Version-1.2) Population-genetics software for exact tests and ecumenicism. J. Hered..

[75] Rice WR (1989). Analyzing tables of statistical tests. Evolution.

[76] Peakall R, Smouse PE (2012). GenAlEx 6.5: genetic analysis in Excel. Population genetic software for teaching and research-an update. Bioinformatics.

[77] FSTAT, a program to estimate and test gene diversities and fixation indices v. 2.9.3 (2002).

[78] Dieringer D, Schlotterer C (2003). MICROSATELLITE ANALYSER (MSA): a platform independent analysis tool for large microsatellite data sets. Mol. Ecol. Notes.

[79] Earl D, vonHoldt B (2012). STRUCTURE HARVESTER: a website and program for visualizing STRUCTURE output and implementing the Evanno method. Conserv Genet Resour..

[80] Pritchard JK, Stephens M, Donnelly P (2000). Inference of population structure using multilocus genotype data. Genetics.

[81] Evanno G, Regnaut S, Goudet J (2005). Detecting the number of clusters of individuals using the software STRUCTURE: a simulation study. Mol. Ecol..

[82] Jakobsson M, Rosenberg NA (2007). CLUMPP: a cluster matching and permutation program for dealing with label switching and multimodality in analysis of population structure. Bioinformatics.

[83] Rosenberg N (2004). DISTRUCT: a program for the graphical display of population structure. Mol. Ecol. Notes.

[84] Pudlo P (2016). Reliable ABC model choice via random forests. Bioinformatics.

[85] Fraimout A (2017). Deciphering the routes of invasion of *Drosophila suzukii* by Means of ABC Random Forest. Mol. Biol. Evol..

[86] Alto BW, Juliano SA (2001). Temperature effects on the dynamics of *Aedes albopictus* (Diptera: Culicidae) populations in the laboratory. J. Med Entomol..

[87] Garza JC, Williamson EG (2001). Detection of reduction in population size using data from microsatellite loci. Mol. Ecol..

[88] Cornuet JM (2008). Inferring population history with DIY ABC: a user-friendly approach to approximate Bayesian computation. Bioinformatics.

[89] Cornuet JM, Ravigné V, Estoup A (2010). Inference on population history and model checking using DNA sequence and microsatellite data with the software DIYABC (v1.0). BMC Bioinforma..

[90] Dubrulle M, Mousson L, Moutailler S, Vazeille M, Failloux AB (2009). Chikungunya virus and Aedes mosquitoes: saliva is infectious as soon as two days after oral infection. PLoS ONE.

[91] FIRTHLOGIT: Stata module to calculate bias reduction in logistic regression v. revised 25 Jul 2015 (Boston College Department of Economics, Boston, MA, USA, 2015).

[92] Adhami J, Reiter P (1998). Introduction and establishment of Aedes (Stegomyia) albopictus skuse (Diptera: Culicidae) in Albania. J. Am. Mosq. Control Assoc..

[93] Sabatini A, Raineri V, Trovato G, Coluzzi M (1990). [*Aedes albopictus* in Italy and possible diffusion of the species into the Mediterranean area]. Parassitologia.

[94] Delaunay P, Jeannin C, Schaffner F, Marty P (2009). [News on the presence of the tiger mosquito *Aedes albopictus* in metropolitan France]. Arch. Pediatr..

[95] Leong M, Grace J (2009). Occurrence and distribution of mosquitoes (Diptera: Culicidae) of public health importance on the Island of Oahu. Proc. Hawaii. Entomol. Soc..

[96] Moore CG, Francy DB, Eliason DA, Monath TP (1988). *Aedes albopictus* in the United States: rapid spread of a potential disease vector. J. Am. Mosq. Control Assoc..

[97] O’Meara GF, Evans LF, Gettman AD, Cuda JP (1995). Spread of *Aedes albopictus* and decline of Ae. aegypti (Diptera: Culicidae) in Florida. J. Med Entomol..

[98] Forattini OP (1986). Identification of *Aedes* (*Stegomyia*) *albopictus* (Skuse) in Brazil. Rev. Saude Publica.

[99] Fé NF, das Graças Vale Barbosa M, Alecrim WD, Guerra MV (2003). [Registration of the occurrence of *Aedes albopictus* in an urban zone in Manaus, Amazonas, Brazil]. Rev. Saude Publica.

[100] Pancetti FG, Honório NA, Urbinatti PR, Lima-Camara TN (2015). Twenty-eight years of *Aedes albopictus* in Brazil: a rationale to maintain active entomological and epidemiological surveillance. Rev. Soc. Bras. Med Trop..

[101] Schweigmann N, Vezzani D, Orellano P, Kuruc J, Boffi R (2004). *Aedes albopictus* in an area of Misiones, Argentina. Rev. Saude Publica.

[102] Ngoagouni C, Kamgang B, Nakouné E, Paupy C, Kazanji M (2015). Invasion of *Aedes albopictus* (Diptera: Culicidae) into central Africa: what consequences for emerging diseases?. Parasit. Vectors.

