# Supplementary material for: Vector competence of Aedes albopictus populations for chikungunya virus is shaped by their demographic history
Source: Commun Biol. 2020 Jun 24;3:326. doi: 10.1038/s42003-020-1046-6 (PMC7314749; doi:10.1038/s42003-020-1046-6)
Supplement: Supplementary file 1 — Supplementary Information [file 42003_2020_1046_MOESM1_ESM.pdf]

Supplementary Table 1 Genetic variability estimates of 25 populations of *Ae. albopictus* from eight regions.

Region	Population	n_a	n_a / n	n_p	n_p / n	A_P	n_r	n_r / n	R_S	H_E
Southeast Asia	JP	4.18	0.32	2	0.15	0.04	11	0.85	2.40	0.43
	CN	3.18	0.32	2	0.20	0.05	0	0.00	2.37	0.46
	TH	5.54	0.18	0	0.00	-	24	0.80	2.56	0.47
Indian Ocean	RE	4.64	0.15	1	0.03	0.05	13	0.43	2.57	0.51
	PROV	4.09	0.14	0	0.00	-	8	0.27	2.41	0.46
	STD	4.27	0.14	0	0.00	-	10	0.33	2.45	0.49
Mediterranean Basin	GR	4.18	0.14	4	0.14	0.02	8	0.28	2.38	0.44
	AL	3.82	0.16	2	0.08	0.06	11	0.48	2.21	0.41
	IT1	4.45	0.14	1	0.03	0.02	14	0.45	2.31	0.42
	IT2	3.27	0.13	0	0.00	-	7	0.28	2.15	0.43
	BL	4.73	0.16	2	0.07	0.07	13	0.43	2.55	0.50
Pacific Ocean	HI	3.18	0.11	0	0.00	-	9	0.31	1.96	0.35
North America	VA	4.73	0.16	3	0.10	0.04	12	0.43	2.48	0.47
	TYS	3.27	0.11	0	0.00	-	8	0.27	2.21	0.44
	VRB	4.00	0.13	0	0.00	-	10	0.33	2.37	0.48
Central America	MXC	3.73	0.12	7	0.23	0.04	7	0.23	2.25	0.45
	PAN	2.54	0.08	0	0.00	-	3	0.10	1.88	0.35
South America	JRB	3.27	0.11	0	0.00	-	6	0.20	2.23	0.45
	MAN	4.36	0.14	0	0.00	-	15	0.50	2.34	0.45
	SAN	3.54	0.12	1	0.03	0.02	7	0.23	2.13	0.41
	PNM	3.27	0.11	0	0.00	-	4	0.13	2.21	0.43
	STR	2.91	0.10	0	0.00	-	7	0.23	1.94	0.36
	MIA	2.18	0.07	0	0.00	-	1	0.03	1.81	0.32
Central Africa	CONG1	4.36	0.14	1	0.03	0.07	11	0.37	2.43	0.47
	CONG2	3.91	0.13	1	0.03	0.02	9	0.30	2.24	0.41

n_a, mean number of alleles; n_a / n, mean number of alleles/individual; n_p, number of private alleles; n_p / n, mean number of private alleles/individual; A_p, mean frequency of private alleles; n_r, number of rare alleles; n_r / n, mean number of rale alleles/individual; R_s, allelic richness; H_E, mean expected heterozygosity

Supplementary Table 2 Pairwise F_{ST} values among wild *Ae. albopictus* populations. F_{ST} values that are underlined are not significantly different from zero ($P > 0.05$) after Bonferroni correction

	JP	CN	TH	RE	PROV	STD	GR	AL	IT1	IT2	BL	HI	VA	TYS	VRB	MXC	PAN	JRB	MAN	SAN	PNM	STR	MIA	CONG1	CONG2
**JP**	0																								
**CN**	<u>0.014</u>	0																							
**TH**	<u>0.034</u>	0.069	0																						
**RE**	0.101	0.091	0.060	0																					
**PROV**	0.095	0.096	<u>0.035</u>	0.057	0																				
**STD**	0.114	0.127	0.077	0.061	<u>0.035</u>	0																			
**GR**	0.134	0.140	0.076	0.129	0.102	0.135	0																		
**AL**	0.069	<u>0.041</u>	0.076	0.094	0.097	0.160	0.186	0																	
**IT1**	0.066	<u>0.047</u>	0.079	0.071	0.097	0.117	0.154	0.084	0																
**IT2**	0.105	0.108	0.069	0.072	0.095	0.106	0.119	0.145	0.081	0															
**BL**	0.104	0.125	0.112	0.102	0.097	0.064	0.178	0.155	0.146	0.132	0														
**HI**	0.146	0.112	0.112	0.125	0.108	0.150	0.165	0.118	0.128	0.120	0.142	0													
**VA**	0.075	0.085	0.056	0.091	0.107	0.122	0.088	0.127	0.113	<u>0.049</u>	0.133	0.114	0												
**TYS**	0.111	0.119	0.124	0.140	0.142	0.122	0.175	0.171	0.179	0.153	0.139	0.167	0.108	0											
**VRB**	0.081	0.067	0.079	0.091	0.087	0.093	0.154	0.121	0.115	0.080	0.084	0.116	0.070	0.065	0										
**MXC**	0.162	0.130	0.134	0.136	0.135	0.164	0.160	0.162	0.139	0.077	0.161	0.135	0.098	0.168	0.107	0									
**PAN**	0.264	0.287	0.201	0.200	0.212	0.195	0.283	0.279	0.250	0.187	0.197	0.282	0.218	0.202	0.110	0.236	0								
**JRB**	0.166	0.161	0.157	0.140	0.162	0.180	0.232	0.143	0.205	0.210	0.149	0.234	0.166	0.176	0.104	0.234	0.233	0							
**MAN**	<u>0.035</u>	<u>0.040</u>	0.053	0.094	0.066	0.098	0.150	0.091	0.079	0.097	0.091	0.146	0.101	0.119	<u>0.047</u>	0.141	0.165	0.103	0						
**SAN**	0.222	0.217	0.189	0.160	0.170	0.173	0.243	0.226	0.229	0.205	0.170	0.275	0.186	0.241	0.148	0.260	0.231	0.115	0.143	0					
**PNM**	0.142	0.168	0.130	0.138	0.159	0.153	0.227	0.169	0.173	0.146	0.126	0.210	0.133	0.211	0.098	0.196	0.174	0.124	0.111	0.112	0				
**STR**	0.324	0.339	0.284	0.232	0.270	0.245	0.350	0.328	0.317	0.317	0.198	0.375	0.307	0.338	0.234	0.371	0.307	0.192	0.239	0.139	0.148	0			
**MIA**	0.258	0.263	0.218	0.218	0.215	0.215	0.307	0.248	0.297	0.301	0.217	0.295	0.243	0.248	0.175	0.331	0.293	0.136	0.175	0.189	0.200	0.230	0		
**CONG1**	0.083	0.082	0.104	0.125	0.126	0.149	0.197	0.122	0.110	0.162	0.148	0.193	0.160	0.144	0.105	0.183	0.208	0.151	<u>0.038</u>	0.230	0.189	0.304	0.244	0	
**CONG2**	0.171	0.178	0.121	0.154	0.112	0.146	0.210	0.175	0.190	0.196	0.162	0.224	0.195	0.172	0.136	0.198	0.223	0.179	0.084	0.256	0.243	0.343	0.273	0.091	0

Supplementary Table 3 Posterior probabilities and 95% confidence intervals for the competing invasion scenarios from the native Southeast Asian area (China, Thailand, Japan) to the newly colonized regions, as estimated by the ABC approach.

**Regions**	**Analysis**	**Scenario**		**Posterior probability**	**Confidence interval**
**La Réunion - Mediterranean basin**			*CN->TH; CN+TH->JP; TH->RE* (Manni et al. 2017)		
	1a	1	CN>TH; CN+TH>JP; TH>RE; JP>PROV	0.0015	[0.0000,0.0965]
		2	CN>TH; CN+TH>JP; TH>RE; CN>PROV	0.0070	[0.0000,0.1014]
		3	CN>TH; CN+TH>JP; TH>RE; TH>PROV	0.0295	[0.0000,0.1220]
		4	CN>TH; CN+TH>JP; TH>RE; RE+TH>PROV	0.2129	[0.0971,0.3286]
		5	CN>TH; CN+TH>JP; TH>RE; RE+CN>PROV	0.1197	[0.0326,0.2067]
		6	CN>TH; CN+TH>JP; TH>RE; RE+JP>PROV	0.0739	[0.0000,0.1615]
		7	CN>TH; CN+TH>JP; TH>RE; CN+JP>PROV	0.0010	[0.0000,0.0960]
		8	CN>TH; CN+TH>JP; TH>RE; JP+TH>PROV	0.0059	[0.0000,0.1004]
		9	CN>TH; CN+TH>JP; TH>RE; TH+CN>PROV	0.0234	[0.0000,0.1160]
		**10**	**CN>TH; CN+TH>JP; TH>RE; RE>PROV**	**0.5252**	**[0.4446,0.6059]**
			CN>AL; JP>VA; VA>IT2; RE+IT2>IT1 (Manni et al. 2017)		
	1b	1	CN>TH; CN+TH>JP; TH>RE; RE>PROV; CN>AL; JP>VA; VA>IT2; RE+IT2>IT1; RE+PROV>BL	0.1281	[0.0000,0.6149]
		2	CN>TH; CN+TH>JP; TH>RE; RE>PROV; CN>AL; JP>VA; VA>IT2; RE+IT2>IT1; RE>BL	0.0023	[0.0000,0.5382]
		3	CN>TH; CN+TH>JP; TH>RE; RE>PROV; CN>AL; JP>VA; VA>IT2; RE+IT2>IT1; PROV>BL	0.0382	[0.0000,0.5551]
		4	CN>TH; CN+TH>JP; TH>RE; RE>PROV; CN>AL; JP>VA; VA>IT2; RE+IT2>IT1; IT1>BL	0.0006	[0.0000,0.5370]
		5	CN>TH; CN+TH>JP; TH>RE; RE>PROV; CN>AL; JP>VA; VA>IT2; RE+IT2>IT1; RE+IT1>BL	0.0268	[0.0000,0.6287]
		**6**	**CN>TH; CN+TH>JP; TH>RE; RE>PROV; CN>AL; JP>VA; VA>IT2; RE+IT2>IT1; PROV+IT1>BL**	**0.8040**	**[0.6827,0.9254]**
**North America**			*CN>TH; CN+TH>JP; JP>HI; JP>VA* (Manni et al. 2017)		
	2a	1	CN>TH; CN+TH>JP; JP>HI; JP>VA; TH>VRB	0.0000	[0.0000,0.0412]
		2	CN>TH; CN+TH>JP; JP>HI; JP>VA; CN>VRB	0.0004	[0.0000,0.0412]
		**3**	**CN>TH; CN+TH>JP; JP>HI; JP>VA; JP>VRB**	**0.4534**	**[0.3707,0.5361]**
		4	CN>TH; CN+TH>JP; JP>HI; JP>VA; HI>VRB	0.0001	[0.0000,0.0412]
		5	CN>TH; CN+TH>JP; JP>HI; JP>VA; VA>VRB	0.0598	[0.0275,0.0921]
		6	CN>TH; CN+TH>JP; JP>HI; JP>VA; HI+VA>VRB	0.0082	[0.0000,0.0473]
		7	CN>TH; CN+TH>JP; JP>HI; JP>VA; JP+HI>VRB	0.1999	[0.1399,0.2599]
		8	CN>TH; CN+TH>JP; JP>HI; JP>VA; JP+VA>VRB	0.2785	[0.2069,0.3501]
	2b	1	CN>TH; CN+TH>JP; JP>HI; JP>VA; JP>VRB; JP>TYS	0.0623	[0.0337,0.0910]
		2	CN>TH; CN+TH>JP; JP>HI; JP>VA; JP>VRB; VRB>TYS	0.3634	[0.3203,0.4065]
		**3**	**CN>TH; CN+TH>JP; JP>HI; JP>VA; JP>VRB; JP+VRB>TYS**	**0.4510**	**[0.4119,0.4900]**
		4	CN>TH; CN+TH>JP; JP>HI; JP>VA; JP>TYS; JP>VRB;	0.0149	[0.0000,0.0475]
		5	CN>TH; CN+TH>JP; JP>HI; JP>VA; JP>TYS; TYS>VRB	0.0364	[0.0052,0.0675]
		6	CN>TH; CN+TH>JP; JP>HI, JP>VA; JP>TYS; JP+TYS>VRB	0.0720	[0.0436,0.1005]

Supplementary Table 3 (continued) Posterior probabilities and 95% confidence intervals for the competing invasion scenarios from the native Southeast Asian area (China, Thailand, Japan) to the newly colonized regions, as estimated by the ABC approach.

Regions	Analysis	Scenario		Posterior probability	Confidence interval
**South America**					
	3a	1	JP>VRB; JP>PNM	0.0858	[0.0634,0.1083]
		2	JP>VRB; JP+VRB>PNM	0.4101	[0.3726,0.4477]
		**3**	**JP>VRB; VRB>PNM**	**0.5040**	**[0.4654,0.5427]**
	3b	1	JP>VRB; VRB>PNM; JP>JRB	0.0013	[0.0000,0.0257]
		2	JP>VRB; VRB>PNM; VRB>JRB	0.1579	[0.1356,0.1801]
		3	JP>VRB; VRB>PNM; PNM>JRB	0.1151	[0.0947,0.1355]
		**4**	**JP>VRB; VRB>PNM; VRB+PNM>JRB**	**0.3540**	**[0.3225,0.3855]**
		5	JP>VRB; VRB>JRB; JRB>PNM	0.1303	[0.1078,0.1528]
		6	JP>VRB; VRB>JRB; VRB+JRB>PNM	0.2415	[0.2073,0.2757]
	3c	**1**	**JP>VRB; VRB>PNM; VRB+PNM>JRB; JRB>MIA**	**0.5165**	**[0.4767,0.5563]**
		2	JP>VRB; VRB>PNM; VRB+PNM>JRB; JP>MIA	0.0002	[0.0000,0.0155]
		3	JP>VRB; VRB>PNM; VRB+PNM>JRB; PNM>MIA	0.0063	[0.0000,0.0208]
		4	JP>VRB; VRB>PNM; VRB+PNM>JRB; VRB>MIA	0.0179	[0.0046,0.0312]
		5	JP>VRB; VRB>PNM; VRB+PNM>JRB; PNM+JRB>MIA	0.1826	[0.1590,0.2063]
		6	JP>VRB; VRB>PNM; VRB+PNM>JRB; VRB+JRB>MIA	0.2428	[0.2116,0.2740]
		7	JP>VRB; VRB>PNM; VRB+PNM>JRB; VRB+PNM>MIA	0.0337	[0.0214,0.0459]
	3d	1	JP>VRB; VRB>PNM; JP>MAN	0.1362	[0.0741,0.1982]
		2	JP>VRB; VRB>PNM; VRB>MAN	0.0636	[0.0000,0.1722]
		3	JP>VRB; VRB>PNM; PNM>MAN	0.0006	[0.0000,0.0816]
		4	JP>VRB; VRB>PNM; JP+PNM>MAN	0.1770	[0.1177,0.2364]
		5	JP>VRB; VRB>PNM; VRB+PNM>MAN	0.0441	[0.0000,0.1207]
		**6**	**JP>VRB; VRB>PNM; JP+VRB>MAN**	**0.5784**	**[0.5330,0.6239]**

Supplementary Table 3 (continued) Posterior probabilities and 95% confidence intervals for the competing invasion scenarios from the native Southeast Asian area (China, Thailand, Japan) to the newly colonized regions, as estimated by the ABC approach.

Regions	Analysis	Scenario		Posterior probability	Confidence interval
**Central America**					
	**4a**	**1**	**JP>VRB; JP+VRB>TYS; VRB>PNM; VRB>MXC**	**0.5680**	**[0.5093,0.6266]**
		2	JP>VRB; JP+VRB>TYS; VRB>PNM; JP>MXC	0.0185	[0.0000,0.0283]
		3	JP>VRB; JP+VRB>TYS; VRB>PNM; PNM>MXC	0.0014	[0.0000,0.0289]
		4	JP>VRB; JP+VRB>TYS; VRB>PNM; VRB+PNM>MCX	0.1811	[0.1443,0.2179]
		5	JP>VRB; JP+VRB>TYS; VRB>PNM; JP+PNM>MXC	0.0205	[0.0041,0.0369]
		6	JP>VRB; JP+VRB>TYS; VRB>PNM; JP+VRB>MXC	0.2185	[0.1788,0.2661]
	4b	1	JP>VRB; JP+VRB>TYS; VRB>PNM; VRB>MXC; JP>PAN	0.0056	[0.0000,0.1229]
		2	JP>VRB; JP+VRB>TYS; VRB>PNM; VRB>MXC; VRB>PAN	0.3235	[0.2396,0.4073]
		3	JP>VRB; JP+VRB>TYS; VRB>PNM; VRB>MXC; PNM>PAN	0.0355	[0.0000,0.1454]
		4	JP>VRB; JP+VRB>TYS; VRB>PNM; VRB>MXC; MXC>PAN	0.0056	[0.0000,0.1234]
		5	JP>VRB; JP+VRB>TYS; VRB>PNM; VRB>MXC; PNM+MXC>PAN	0.0077	[0.0000,0.1913]
		6	JP>VRB; JP+VRB>TYS; VRB>PNM; VRB>MXC; VRB+MXC>PAN	0.0586	[0.0000,0.2057]
		**7**	**JP>VRB; JP+VRB>TYS; VRB>PNM; VRB>MXC; VRB+PNM>PAN**	**0.4836**	**[0.4020,0.5652]**
**Central Africa**					
	5	1	JP>VRB; VRB>PNM; JP+VRB>MAN; JP>CONG1	0.0011	[0.0000,0.1900]
		2	JP>VRB; VRB>PNM; JP+VRB>MAN; VRB>CONG1	0.0001	[0.0000,0.1890]
		3	JP>VRB; VRB>PNM; JP+VRB>MAN; JP+VRB>CONG1	0.0047	[0.0000,0.1933]
		4	JP>VRB; VRB>PNM; JP+VRB>MAN; JP+MAN>CONG1	0.0887	[0.0000,0.2687]
		**5**	**JP>VRB; VRB>PNM; JP+VRB>MAN; MAN>CONG1**	**0.8602**	**[0.8304,0.8899]**
		6	JP>VRB; VRB>PNM; JP+VRB>MAN; VRB+MAN>CONG1	0.0451	[0.0000,0.2429]

Supplementary Table 4 Vector competence data used in the present study

Country	Population	Population code	N	Viral strain	CHIKV genotype	BM titre	dpi	DE (%)	TE (%)
La Réunion	Providence	PROV	19	CHIKV_0621	ECSA E1-226V	7.5	6		89.5
	Providence	PROV	20	CHIKV_0621	ECSA E1-226V	7.5	7		80.0
Italy	Cesena	IT1	40	CHIKV_0621	ECSA E1-226V	7.5	6	97.5	80.0
	Cesena	IT1	40	CHIKV_1909	ECSA	7.5	6	100.0	50.0
France	Bar sur Loup	BL	13	CHIKV_0621	ECSA E1-226V	7.5	6	100.0	61.5
U.S.A.	St Louis (Missouri)	TYS	30	CHIKV_0621	ECSA E1-226V	7.5	7	96.7	56.7
	St Louis (Missouri)	TYS	30	CHIKV_0621	ECSA E1-226V	7.5	10	93.3	40.0
	St Louis (Missouri)	TYS	30	CHIKV_05115	ECSA	7.5	7	83.3	50.0
	St Louis (Missouri)	TYS	11	CHIKV_05115	ECSA	7.5	10	63.6	27.3
	Vero Beach (Florida)	VRB	30	CHIKV_0621	ECSA E1-226V	7.5	7	93.3	26.7
	Vero Beach (Florida)	VRB	7	CHIKV_0621	ECSA E1-226V	7.5	10	85.7	14.3
	Vero Beach (Florida)	VRB	30	CHIKV_05115	ECSA	7.5	7	73.3	26.7
	Vero Beach (Florida)	VRB	30	CHIKV_05115	ECSA	7.5	10	96.7	16.7
Mexico	Tapachula	MXC	30	CHIKV_0621	ECSA E1-226V	7.5	7	73.3	16.7
	Tapachula	MXC	30	CHIKV_0621	ECSA E1-226V	7.5	10	70.0	20.0
	Tapachula	MXC	30	CHIKV_05115	ECSA	7.5	7	73.3	23.3
	Tapachula	MXC	30	CHIKV_05115	ECSA	7.5	10	53.3	23.3
Panama	Colón	PAN	30	CHIKV_0621	ECSA E1-226V	7.5	7	96.7	83.3
	Colón	PAN	30	CHIKV_0621	ECSA E1-226V	7.5	10	96.7	73.3
	Colón	PAN	30	CHIKV_05115	ECSA	7.5	7	93.3	33.3
	Colón	PAN	30	CHIKV_05115	ECSA	7.5	10	83.3	76.7
	Colón	PAN	30	CHIKV_NC	ASIA	7.5	7	96.7	73.3
	Colón	PAN	30	CHIKV_NC	ASIA	7.5	10	96.7	46.7
Brazil	Jurujuba	JRB	30	CHIKV_0621	ECSA E1-226V	7.5	7	100.0	96.7
	Jurujuba	JRB	30	CHIKV_0621	ECSA E1-226V	7.5	10	100.0	76.7

Supplementary Table 4 (continued) Vector competence data used in the present study

Country	Population	Population code	N	Viral strain	CHIKV genotype	BM titre	dpi	DE (%)	TE (%)
Brazil	Manaus	MAN	30	CHIKV_0621	ECSA E1-226V	7.5	7	96.7	73.3
	Manaus	MAN	36	CHIKV_0621	ECSA E1-226V	7.5	10	100.0	69.4
	Manaus	MAN	31	CHIKV_05115	ECSA	7.5	7	90.3	35.5
	Manaus	MAN	34	CHIKV_05115	ECSA	7.5	10	97.1	52.9
	Manaus	MAN	30	CHIKV_NC	ASIA	7.5	7	90.0	43.3
	Manaus	MAN	30	CHIKV_NC	ASIA	7.5	10	93.3	53.3
	Santos	SAN	30	CHIKV_0621	ECSA E1-226V	7.5	7	100.0	43.3
	Santos	SAN	30	CHIKV_0621	ECSA E1-226V	7.5	10	100.0	50.0
	Santos	SAN	8	CHIKV_05115	ECSA	7.5	7	87.5	62.5
	Parnamirim	PNM	30	CHIKV_0621	ECSA E1-226V	7.5	7	93.3	66.7
	Parnamirim	PNM	30	CHIKV_0621	ECSA E1-226V	7.5	10	90.0	66.7
	Santarém	STR	29	CHIKV_0621	ECSA E1-226V	7.5	7	100.0	58.6
	Santarém	STR	20	CHIKV_0621	ECSA E1-226V	7.5	10	100.0	55.0
	Santarém	STR	26	CHIKV_05115	ECSA	7.5	7	88.5	50.0
Argentina	Eldorado (Misiones)	MIA	30	CHIKV_0621	ECSA E1-226V	7.5	7	60.0	36.7
	Eldorado (Misiones)	MIA	30	CHIKV_0621	ECSA E1-226V	7.5	10	93.3	60.0
	Eldorado (Misiones)	MIA	30	CHIKV_05115	ECSA	7.5	7	66.7	76.7
	Eldorado (Misiones)	MIA	30	CHIKV_05115	ECSA	7.5	10	80.0	40.0
	Eldorado (Misiones)	MIA	30	CHIKV_NC	ASIA	7.5	7	93.3	30.0
	Eldorado (Misiones)	MIA	30	CHIKV_NC	ASIA	7.5	10	96.7	46.7
Congo	Brazzaville	CONG1	39	CHIKV_DRC	ECSA	6.5	7	20.5	15.4
	Brazzaville	CONG1	26	CHIKV_Congo	ECSA E1-226V	6.5	7	53.8	30.8

N: number of mosquitoes analyzed. BM: blood meal. dpi: day post-infection. Dissemination efficiency (DE) and transmission efficiency (TE) respectively refer to the proportion of mosquitoes with infectious viral particles in the head and in the saliva among the number of mosquitoes tested. ECSA E1-226V: strains from East-Center and South African genotype with E1-226V substitution. ECSA: strains from East-Center and South African genotype with E1-226A substitution. ASIA: strains from Asian genotype with E1-226A substitution.

Supplementary Table 5 Factors associated with CHIKV dissemination in *Ae. albopictus* populations /lineages (univariate analysis)

Factor	Variable	N	N (%) disseminated	Crude OR (95% CI)[1]	P
**Days post infection**	6-7	762	645 (84.6)	1	**0.0049**
	10	528	475 (90.0)	1.63 (1.15 - 2.30)	
**Virus genotype**[2]	ASIA	180	170 (97.4)	5.22 (2.67 - 10.23)	**<0.0001**
	ECSA	485	371 (76.5)	1	
	ECSA E1-226V	625	579 (92.6)	3.87 (2.68 - 5.58)	
**Blood meal titre**	6.5	65	22 (33.8)	0.06 (0.03 - 0.10)	**<0.001**
	7.5	1 225	1 098 (89.6)	1	
**Main *K*-ancestry**	1	443	401 (90.5)	1.66 (1.11 - 2.48)	**0.0001**
	2	13	13 (100)	4.74 (0.28 - 80.57)	
	3	498	424 (85.1)	1	
	4	-	-	-	
	5	256	203 (79.3)	0.67 (0.45 - 0.98)	
	6	80	79 (98.7)	9.30 (1.82 - 47.66)	
***K*1 ancestry**	≤0.25	847	719 (84.9)	1	**0.004**
	>0.25	443	401 (90.5)	1.70 (1.17 - 2.46)	
***K*2 ancestry**	≤0.25	1277	1107 (86.7)	1	0.24
	>0.25	13	13 (100)	2.80 (0.46 - Inf)	
***K*3 ancestry**	≤0.25	732	641 (87.6)	1	0.36
	>0.25	558	479 (85.8)	0.86 (0.63 - 1.19)	
***K*5 ancestry**	≤0.25	1034	917 (88.7)	1	**<0.001**
	>0.25	256	203 (79.3)	0.49 (0.34 - 0.70)	
***K*6 ancestry**	≤0.25	1210	1041 (86.0)	1	**<0.001**
	>0.25	80	79 (98.7)	12.82 (1.77 - 92.80)	
***K*-ancestry profile**	*K*1, *K*5 and *K*6 ≤0.25	511	437 (85.5)	1	**<0.001**
	Only *K*1>0.25	443	401 (90.5)	1.62 (1.08 - 2.42)	
	Only *K*5>0.25	256	203 (79.3)	0.65 (0.44 - 0.96)	
	Only *K*6>0.25	80	79 (98.7)	13.38 (1.83 - 97.64)	

[1]OR : Odds Ratio ; CI : confidence interval

[2]ASIA: CHIKV strains from Asian genotype. ECSA: CHIKV strains from East-Central-South African genotype harbouring an alanine at position 226 of E1 glycoprotein. ECSA E1-226V: CHIKV strains from East-Central-South African genotype harbouring a valine at position 226 of E1 glycoprotein.

Supplementary Table 6 Factors associated with CHIKV transmission in *Ae. albopictus* populations /lineages (univariate analysis)

Factor	Variable	N	N (%) transmission	Crude OR (95% CI)[1]	*P*
**Days post infection**	6-7	684	407 (59.5)	1	0.210
	10	475	265 (55.8)	0.86 (0.68 - 1.09)	
**Virus genotype**[2]	ASIA	170	88 (51.8)	1.01 (0.70 - 1.45)	**0.0002**
	ECSA	371	191 (51.5)	1	
	ECSA E1-226V	618	393 (63.6)	**1.65 (1.27 - 2.14)**	
**Blood meal titre**	6.5	22	14 (63.6)	1.27 (0.53 - 3.06)	0.590
	7.5	1137	658 (57.9)	1	
**Main *K*-ancestry**	1	401	250 (62.3)	1.69 (1.28 - 2.23)	**<0.0001**
	2	52	41 (78.8)	3.80 (1.90 - 7.59)	
	3	424	210 (49.5)	1	
	4	-	-	-	
	5	203	119 (58.6)	1.44 (1.03 - 2.02)	
	6	79	52 (65.8)	1.96 (1.19 - 3.24)	
***K*1 ancestry**	≤0.25	758	422 (55.7)	1	**0.0280**
	>0.25	401	250 (62.3)	1.32 (1.03 - 1.69)	
***K*2 ancestry**	≤0.25	1107	631 (57.0)	1	**0.0012**
	>0.25	52	41 (78.8)	2.81 (1.43 - 5.52)	
***K*3 ancestry**	≤0.25	680	422 (62.0)	1	**0.0008**
	>0.25	479	250 (52.1)	0.67 (0.53 - 0.85)	
***K*5 ancestry**	≤0.25	956	553 (57.8)	1	0.840
	>0.25	203	119 (58.6)	1.03 (0.76 - 1.41)	
***K*6 ancestry**	≤0.25	1080	620 (57.4)	1	0.140
	>0.25	79	52 (65.8)	1.43 (0.88 - 2.30)	
***K*-ancestry profile**	K2 and K3 ≤0.25	628	381 (60.7)	1	**0.0001**
	Only K2>0.25	52	41 (78.8)	2.42 (1.22 - 4.79)	
	Only K3>0.25	479	250 (52.2)	0.71 (0.56 - 0.90)	

[1]OR: Odds Ratio; CI: confidence interval

[2]ASIA: CHIKV strains from Asian genotype. ECSA: CHIKV strains from East-Central-South African genotype harbouring an alanine at position 226 of E1 glycoprotein. ECSA E1-226V: CHIKV strains from East-Central-South African genotype harbouring a valine at position 226 of E1 glycoprotein.

Supplementary Table 7 Definition and prior distribution of parameters used in the ABC analyses for describing the set of scenarios investigated for the reconstruction of the invasion of *Ae. albopictus*

Parameter	Distribution	Interval
Effective population size N_i ($_i$ = population of interest)	Uniform	500-100000
Number of founder events, NF_i	Uniform	1-100
Bottleneck duration, Db_i	Uniform	0-50
Time of events (in generations backward in time):		
Founder event in population CN	Uniform	3000-80000
Founder event in population TH	Uniform	3000-80000
Founder event in population JP	Uniform	3000-80000
Founder event in population RE	Uniform	2000-6000
Founder event in population PROV	Uniform	2000-6000
Founder event in population HI	Uniform	1300-2000
Founder event in population VA	Uniform	300-650
Founder event in population VRB	Uniform	300-650
Founder event in population TYS	Uniform	100-650 (4-17 generations/year)
Founder event in population PNM	Uniform	250-600
Founder event in population JRB	Uniform	250-600
Founder event in population MXC	Uniform	250-500
Founder event in population MIA	Uniform	150-350
Founder event in population AL	Uniform	100-350 (4-7 generations/year)
Founder event in populations IT1/IT2	Uniform	50-300 (4-7 generations/year)
Founder even in population BL	Uniform	50-250 (4-7 generations/year)
Founder event in population PAN	Uniform	100-250
Founder event in population MAN	Uniform	100-300
Founder event in population CONG	Uniform	5-100
Rates of admixture (ra)	Uniform	0.001-0.999
Mutation model parameters:		
Mean mutation rate	Uniform	10^{-5}-10^{-3}
Mean parameter of the geometric distribution of repeats, *P*	Gamma	0.1-0.3
Mean single nucleotide insertion/deletion mutation rate, SNI	Log-uniform	10^{-8}-10^{-5}
