# Supplementary material for: Vector competence of Aedes albopictus populations for chikungunya virus is shaped by their demographic history
Source: Commun Biol. 2020 Jun 24;3:326. doi: 10.1038/s42003-020-1046-6 (PMC7314749; doi:10.1038/s42003-020-1046-6)
Supplement: Supplementary file 3 — Description of Additional Supplementary Files [file 42003_2020_1046_MOESM3_ESM.pdf]

Description of Additional Supplementary Files

File Name: **Supplementary Data 1**
Description: Individual coefficient of ancestry obtained from a STRUCTURE run with *K* = 6 for 702 individuals of *Ae. albopictus* from 25 samples collected in eight different geographical areas.
