# Supplementary material for: Vector competence of Aedes albopictus populations for chikungunya virus is shaped by their demographic history
Source: Commun Biol. 2020 Jun 24;3:326. doi: 10.1038/s42003-020-1046-6 (PMC7314749; doi:10.1038/s42003-020-1046-6)
Supplement: Supplementary file 4 — Reporting Summary [file 42003_2020_1046_MOESM4_ESM.pdf]

nature research

Corresponding author(s): Anna R Malacrida

Last updated by author(s): May 22, 2020

Reporting Summary

Nature Research wishes to improve the reproducibility of the work that we publish. This form provides structure for consistency and transparency in reporting. For further information on Nature Research policies, see Authors & Referees and the Editorial Policy Checklist.

Statistics

For all statistical analyses, confirm that the following items are present in the figure legend, table legend, main text, or Methods section.

n/a	Confirmed	
☐	☒	The exact sample size (*n*) for each experimental group/condition, given as a discrete number and unit of measurement
☐	☒	A statement on whether measurements were taken from distinct samples or whether the same sample was measured repeatedly
☐	☒	The statistical test(s) used AND whether they are one- or two-sided<br>*Only common tests should be described solely by name; describe more complex techniques in the Methods section.*
☐	☒	A description of all covariates tested
☐	☒	A description of any assumptions or corrections, such as tests of normality and adjustment for multiple comparisons
☐	☒	A full description of the statistical parameters including central tendency (e.g. means) or other basic estimates (e.g. regression coefficient) AND variation (e.g. standard deviation) or associated estimates of uncertainty (e.g. confidence intervals)
☐	☒	For null hypothesis testing, the test statistic (e.g. *F*, *t*, *r*) with confidence intervals, effect sizes, degrees of freedom and *P* value noted<br>*Give P values as exact values whenever suitable.*
☐	☒	For Bayesian analysis, information on the choice of priors and Markov chain Monte Carlo settings
☐	☒	For hierarchical and complex designs, identification of the appropriate level for tests and full reporting of outcomes
☒	☐	Estimates of effect sizes (e.g. Cohen's *d*, Pearson's *r*), indicating how they were calculated

Our web collection on statistics for biologists contains articles on many of the points above.

Software and code

Policy information about availability of computer code

Data collection	*Provide a description of all commercial, open source and custom code used to collect the data in this study, specifying the version used OR state that no software was used.*
Data analysis	TANDEM v1.09; GENEPOP V. 4.2; GenAlEx 6.5; FSTAT V.2.9.3.2; Microsatellite Analyzer (MSA) V.4.05; STRUCTURE V 2.3.2; STRUCTURE HARVESTER; CLUMPP; DISTRUCT; DIYABC v2.1;

For manuscripts utilizing custom algorithms or software that are central to the research but not yet described in published literature, software must be made available to editors/reviewers. We strongly encourage code deposition in a community repository (e.g. GitHub). See the Nature Research guidelines for submitting code & software for further information.

Data

Policy information about availability of data

All manuscripts must include a data availability statement. This statement should provide the following information, where applicable:

- Accession codes, unique identifiers, or web links for publicly available datasets
- A list of figures that have associated raw data
- A description of any restrictions on data availability

The raw genotype data has been submitted to the Open Science Framework data repository. All other relevant data are within the paper and its Supporting Information files. Accession code: https://osf.io/g5pt3/?view_only=ee95053bf7cf496292320e11df0476a8

Field-specific reporting

Please select the one below that is the best fit for your research. If you are not sure, read the appropriate sections before making your selection.

☒ Life sciences ☐ Behavioural & social sciences ☐ Ecological, evolutionary & environmental sciences

For a reference copy of the document with all sections, see nature.com/documents/nr-reporting-summary-flat.pdf

Life sciences study design

All studies must disclose on these points even when the disclosure is negative.

Sample size	Sample size of mosquito population samples were determined (see Table 1). We used 11 highly informative SSR loci applied to 702 individuals from 25 localities to produce a SSR genotyping dataset with no major bias in statistical analyses
Data exclusions	No data was excluded
Replication	Genotype at the 11 loci was replicated for each individual. When microsatellite amplification was not successful, or allele scoring was unclear, a new DNA extraction was performed
Randomization	individuals were grouped according to their population.
Blinding	Investigators were blinded to group allocation during data collection and analysis

Reporting for specific materials, systems and methods

We require information from authors about some types of materials, experimental systems and methods used in many studies. Here, indicate whether each material, system or method listed is relevant to your study. If you are not sure if a list item applies to your research, read the appropriate section before selecting a response.

Materials & experimental systems

n/a	Involved in the study	
☒	☐	Antibodies
☒	☐	Eukaryotic cell lines
☒	☐	Palaeontology
☐	☒	Animals and other organisms
☒	☐	Human research participants
☒	☐	Clinical data

Methods

n/a	Involved in the study	
☒	☐	ChIP-seq
☒	☐	Flow cytometry
☒	☐	MRI-based neuroimaging

Animals and other organisms

Policy information about studies involving animals; ARRIVE guidelines recommended for reporting animal research

Laboratory animals	No laboratory animals were used
Wild animals	field collected mosquitoes collected as eggs in ovitraps.
Field-collected samples	Field collected samples were maintained in 30x30x30 cm cages at 26°C with 70% relative humidity and a 12:12 h (light: dark) photoperiod. They were fed with 20% sucrose solution. Females were females were fed on commercial blood using an artificial feeding system (Hemotek Ltd®)
Ethics oversight	This study did not include the use of humans or other vertebrate animals. No ethics approval is required for experimentation of the study organism, Aedes albopictus. The insect samples were received in accordance with European Union rules (Directive 97/78/CE).

Note that full information on the approval of the study protocol must also be provided in the manuscript.
