# Supplementary material for: Vector competence of Aedes albopictus populations for chikungunya virus is shaped by their demographic history
Source: Commun Biol. 2020 Jun 24;3:326. doi: 10.1038/s42003-020-1046-6 (PMC7314749; doi:10.1038/s42003-020-1046-6)
Supplement: Supplementary file 5 — Peer Review File [file 42003_2020_1046_MOESM5_ESM.pdf]

Reviewers' comments:

Reviewer #1 (Remarks to the Author):

Refer to attached file.

Reviewer #2 (Remarks to the Author):

This paper reports an analysis of 11 microsatellites that have been genotyped in a large collection of mosquitoes. This is used to reconstruct the dispersal of the mosquitoes round the world. The vector competence of mosquitoes was then measured and linked to the genetic data. Given the importance of this species these results are of general interest, and the link to vector competence was novel.

A particularly curious feature of the paper is that you can read the abstract, introduction, results, discussion and conclusion without knowing what data is being analysed. In an era when large genomic datasets are common, this left me wondering whether this was intentional. Regardless, it makes it very hard to understand the results. The fact that this is an analysis of 11 microsatellite loci should be stated in the abstract and the first line of the results.

The most novel aspect of this work was to link the demographic history of populations to their vector competence. The analysis that was performed appears to show little more than that there is geographical variation in vector competence. As nearby populations tend to be genetically similar, any geographical structure in phenotype will correlate with genotype. Geographical structure could arise for reasons other than shared ancestry, such as adaptation to local conditions. What would be considerably more interesting would be to show that genetic ancestry predicts vector competence after controlling for geography. This could easily be achieved by adding a measure of geographical proximity to the statistical model. I am sure there are more elegant ways to approach this too.

The way that the ancestry components were included in the analysis of viral titres seems rather adhoc. Why consider just one or two of the components. I can see no reason why this is done. I would suggest that the 6 ancestry components are added to the model as fixed effects. It would still be possible to test the overall significance of genetic ancestry. I found the description of these statistics in the methods hard to follow. Please add a formal equation that describes the model used.

The interpretation of the ancestry fractions is very literal, despite this species clearly having undergone changes in population size that are known to confound these analyses. These issues are described in a paper by Daniel Falush (A tutorial on how not to over-interpret STRUCTURE and ADMIXTURE bar plots, Nature Coomunication), and their effects in a related species of mosquito are discussed in Crawford et al, BMC Biology. Throughout the manuscript the literal interpretation of these plots as a set of ancestral populations should be avoided in the absence of supporting information. This may require rewriting significant sections of text.

The ABC analyses are the second approach taken to demographic reconstruction. In the main text there is no indication of statistical uncertainty, which made it very hard to interpret these results. The supplementary table suggests that many of the relationships are well supported (they are found across all the well-supported models). It would be helpful to show this information on the figure in some way to highlight poorly supported relationships. A second source of uncertainty is the long list of assumptions being made – priors, changes in Ne, mutation rates etc. It is very hard to understand if altering these alters the outcome. It would be helpful to explore some of these issues, perhaps with a supplementary results that repeat the analyses with different assumptions. This, together with the

comments in the preceding paragraph, left me uncertain how robust the scenario in the summary figure is.
The prose in the results section was hard to read and follow as it tended to involve a very detailed repetition of information in figures and tables. To understand the major conclusions I found it simpler to look at the images. I would suggest simplifying the text.

A few minor comments:
1. The word 'variation' should not be used to replace 'variance' (eg line 99). The former has no precise statistical meaning.
2. I struggled to follow much of the first paragraph which is largely describing Fst. For example, this sentence 'The most homogeneous area was the ancestral Southeast Asia, with populations that maintained low levels of differentiation with La Réunion, the Mediterranean Basin, Hawaii and North America' homogenous needs defining (Fst I guess), and some numbers put on how this conclusion is reached. The density of geographical sampling seems to affect this result. Similarly 'Levels of structuring were observed within La Réunion, while the Mediterranean area appears to be heterogeneous.' Means little to me. Fst within le reunion looks rather low to me. Overall, this paragraph could do with simplifying to get to the take-home message – the detailed description of the figure seemed less informative than looking at the picture
3. Maybe Table 2 could go as a supplement? Fig 2 seems to convey this information more effectively
4. Lines 123-139 again describes a lot of results. I was left looking at the figure in order to conclude the results are really rather interesting. Some rewriting could make the prose much more impactful – scrap the names of ancestry components and other details that are all in Tables and Figures, and instead lead with the biological insight. The different ancestry of relatively nearby populations is very striking.
5. Define abbreviations at first use (eg DE). Reducing the use of abbreviations aids readability.
6. Line 207. It reads very oddly not to mention what the experiment was here. There seems to be a presumption the methods are read first.

Worldwide invasion dynamics of *Aedes albopictus* shaped the vector competence of its populations for chikungunya virus

Vega-Rúa et al.

This study provided a comprehensive analysis of relationships between *Ae. albopictus* mosquito ancestry and vector competence for chikungunya virus. The genetic analyses of mosquito populations are appropriate and provide additional evidence for geographic origins following invasion. This study provides a quantitative assessment of variation in vector competence by geographic origin of a mosquito population, in particular, degree of ancestry within a genetic lineage. Additional details of methods of the vector competence are needed. Also, rapid disseminated infection of chikungunya virus in *Ae. albopictus* (previously established) hinders the ability to determine population-dependent differences in disseminated infection (especially decreased dissemination) when assayed at later times during incubation (7 and 10 days) following ingestion of the infected blood meal. Throughout the paper, vector competence is used interchangeable with "ability to transmit chikungunya virus." However, vector competence is a weak contributor to vectorial capacity, the latter index that is more relevant to public health concerns.

<u>Abstract.</u>

Lines 27-29. Suggested rephrasing sentence. "…health concerns as it is one of the main vectors of…"

Lines 30-34. The ability of *Ae. albopictus* populations to transmit CHIKV includes other parameters than vector competence.

<u>Introduction.</u>

Lines 52-54. I suggest including another relevant reference.

Juliano SA, Lounibos LP. 2005. Ecology of invasive mosquitoes: effects on resisent species and on human health. Ecol Lett 8: 558-574.

Lines 54-57. Specify whether vector competence was demonstrated in nature or through laboratory infection studies.

Lines 57-59. Please support this statement evidence from the literature. The reference included here refers to *A. aegypti*.

Lines 66-69. This statement does not seem consistent with the widespread cases of chikungunya fever in the Americas attributable to the Asian lineage of chikungunya virus (Lanciotti and Lambert 2016). The Asian lineage was the prevalent genotype in this region.

Lanciotti RS, Lambert AJ. 2016. Phylogenetic analysis of chikungunya virus strains circulating in Western Hemisphere. Am J Trop Med Hyg 94: 800-803.

Lines 76-78. Vector competence is a weak contributor to risk of transmission (e.g., vectorial capacity).

Some parameters are more sensitive to changing vectorial capacity than others (parameters that are squared and exponential contribute more strongly to determining vectorial capacity) (Lounibos and Kramer 2016). It would be appropriate to include this information.

Lounibos LP, Kramer LD. 2016. Invasiveness of *Aedes aegypti* and *Aedes albopictus* and vectorial capacity for chikungunya virus. J. Infect. Dis. 214 (suppl 5): S453-S458.

<u>Results</u>

Lines 98-101. These results seems unexpected. I would expect greater variation between geographic populations that, presumably, have low (negligible) gene flow between populations.

Lines 169-171. *Aedes albopictus* was first recognized to establish a persistent population in Texas.

The description of relationships and origins of populations seem adequate.

Lines 207-209. The baseline high levels of disseminated infection hamper the ability to observe genetic factors may enhance disseminated infection. In contrast, the ability to assess genetic-associated reductions in disseminated infection is feasible.

<u>Discussion</u>

Overall, this section is well written.

Lines 359-363. Susceptibility to infection was not measured in this study (i.e., midgut infection barrier(s)).

Lines 391-393. Rephrase for clarity.

It would be helpful to describe limitations of vector competence measurements in determining a mosquitoes ability to transmit a pathogen. That is, several other factors interact to determine the probability of an infectious bite is received by a susceptible host.

<u>Material and Methods</u>

Lines 471-473. Please provide information on the numbers of founding individuals from the field that were used to establish laboratory colonies. According to Table 1, very few founding individuals comprised the establishing laboratory population. This approach brings into question whether populations went through a bottleneck which may result in founder effects.

Please specify the generation tested for the genetic and vector competence assays. Many collections were made in 2010-2013. Depending on when the tests were performed, these mosquitoes have been maintained in laboratory conditions for numerous generations and may not be representative of field populations.

Lines 478-480. This approach seems like a good strategy to address the focus of the study.

Lines 492-494. It seems appropriate to cite the following paper to support this statement;

Chen, X-G, et al. 2015. Genome sequence of the Asian tiger mosquito *Aedes albopictus*, reveals insights into its biology, genetics, and evolution. Proc Natl Acad Sci USA 112(44):E5907-E5915.

Line 501. "…well-documented…"

The genetic diversity analyses, population structure, and analyses of demographic history are appropriate and comprehensive.

Lines 574-576. This explanation is helpful to the reader.

Line 587. Please provide additional details on how the vector competence experiments were performed (e.g., how was saliva collected?). When were mosquitoes tested for disseminated infection and saliva infection? Were mosquitoes tested at more than one time period following ingestion of CHIKV infected blood?

What was the viral titer in the infected bloodmeal? Was it representative of viremia in humans. The viral dose will influence disseminated infection and saliva infection.

Lines 588-589. Please specify generation number for each population.

Lines 594-596. Transmission efficiency suggests the occurrence of a transmission event (e.g., passage of pathogen to a susceptible vertebrate host). Saliva infection (or transmission potential) seems like a better descriptor.

Response to the Editor

Manuscript Title: "Worldwide invasion dynamics of *Aedes albopictus* shaped the vector competence of its populations for chikungunya virus"

On behalf of all co-authors, I thank the reviewers for their constructive remarks that contributed to improve the quality of our manuscript. The manuscript was amended following the reviewer's instructions, with changes highlighted in the text. Line numbers referenced in our comments correspond to the original version of the manuscript.

Reviewers' comments:

Reviewer 1 (Remarks to the Author):
This study provided a comprehensive analysis of relationships between *Ae. albopictus* mosquito ancestry and vector competence for chikungunya virus. The genetic analyses of mosquito populations are appropriate and provide additional evidence for geographic origins following invasion. This study provides a quantitative assessment of variation in vector competence by geographic origin of a mosquito population, in particular, degree of ancestry within a genetic lineage. Additional details of methods of the vector competence are needed. Also, rapid disseminated infection of chikungunya virus in *Ae. albopictus* (previously established) hinders the ability to determine population-dependent differences in disseminated infection (especially decreased dissemination) when assayed at later times during incubation (7 and 10 days) following ingestion of the infected blood meal. Throughout the paper, vector competence is used interchangeable with "ability to transmit chikungunya virus." However, vector competence is a weak contributor to vectorial capacity, the latter index that is more relevant to public health concerns.

Reply: Many thanks for this general comment that we took into account for the manuscript revisions. It is true that the data available for our dissemination analysis had a high baseline given the late days post-infection in which they were taken for CHIKV (7 and 10 days). The nature of these data could possibly make undetectable dissemination differences at finer ancestry and geographic scales that could be detected if data were collected at earlier days post-infection (i.e. day 3). However, at earlier days post-infection we can detect differences that may disappear at later dpi when the dissemination profiles reach their maximum values. Here, we were able to detect dissemination differences according to population's ancestry at broader scales. The fact of finding significant dissemination differences that were still visible at such late days post-infection, where the maximum of dissemination that could be sustained by the *Ae. albopictus* populations is reached according to most of CHIKV kinetics published (Vega Rua et al, 2014, Dubrulle et al 2009), highlights their importance and persistence.
Regarding the differences between vectorial capacity and vector competence, we agree with the reviewer and we made changes throughout the manuscript to increase the precision of the statements. It is true that when you look pragmatically the vectorial capacity equation, other parameters such as extrinsic incubation period or longevity impact more the pathogen transmission when compared to vector competence. While the other parameters such longevity, density etc are subject to significant variations, difficult to predict, vector competence is measured using a same experimental design making comparisons possible.

Reviewer 1: Abstract - Lines 27-29. Suggested rephrasing sentence. "…health concerns as it is one of the main vectors of…"

Reply: The sentence has been rephrased as suggested

Reviewer 1: Lines 30-34. The ability of *Ae. albopictus* populations to transmit CHIKV includes other parameters than vector competence.

Reply: replaced by "which influenced their vector competence for CHIKV".

Reviewer 1: Introduction Lines 52-54. I suggest including another relevant reference.
Juliano SA, Lounibos LP. 2005. Ecology of invasive mosquitoes: effects on resident species and on human health. Ecol Lett 8: 558-574.

Reply: The reference has been added in the mentioned statement

Reviewer 1: Lines 54-57. Specify whether vector competence was demonstrated in nature or through laboratory infection studies.

Reply: sentence rephrased: "…as experimental assessments have shown the species' competence for at least 20 arboviruses…"

Reviewer 1: Lines 57-59. Please support this statement evidence from the literature. The reference included here refers to *A. aegypti*.

Reply: Many thanks for this comment. The reference was replaced by:

1. Kraemer MUG, Reiner RC, Brady OJ, Messina JP, Gilbert M, Pigott DM, et al. Past and future spread of the arbovirus vectors Aedes aegypti and Aedes albopictus. Nat Microbiol. Springer US; 2019;4. doi:10.1038/s41564-019-0376-y

2. Cunze S, Kochmann J, Koch LK, Klimpel S. Niche conservatism of Aedes albopictus and Aedes aegypti - Two mosquito species with different invasion histories. Sci Rep. 2018;8: 1–10. doi:10.1038/s41598-018-26092-2

Reviewer 1: Lines 66-69. This statement does not seem consistent with the widespread cases of chikungunya fever in the Americas attributable to the Asian lineage of chikungunya virus (Lanciotti and Lambert 2016). The Asian lineage was the prevalent genotype in this region.

Lanciotti RS, Lambert AJ. 2016. Phylogenetic analysis of chikungunya virus strains circulating in Western Hemisphere. Am J Trop Med Hyg 94: 800-803.

Reply: The sentence was rephrased for clarity: "comprises three genetic lineages: Asian, West African and East/Central/South African (ECSA) which apparently evolved independently in different geographic areas"

Reviewer 1: Lines 76-78. Vector competence is a weak contributor to risk of transmission (e.g., vectorial capacity).
Some parameters are more sensitive to changing vectorial capacity than others (parameters that are squared and exponential contribute more strongly to determining vectorial capacity) (Lounibos and Kramer 2016). It would be appropriate to include this information.

Lounibos LP, Kramer LD. 2016. Invasiveness of *Aedes aegypti* and *Aedes albopictus* and vectorial capacity for chikungunya virus. J. Infect. Dis. 214 (suppl 5): S453-S458.

Reply: We replaced “viral competence” by “vectorial capacities” and we added the citation of the suggested paper. Also, we mentioned the importance of other vectorial capacity contributors in several parts of the discussion. For instance:

- “Although other factors influencing vectorial capacity may interact and determine that an infectious bite is received by a susceptible human host”,

- “Beyond vector competence, context-specific differences on parameters impacting more importantly the vectorial capacity (i.e. longevity, host feeding) (Lounibos and Kramer 2016) may challenge risk prediction for arboviruses and should be taken into account in further studies.”

Reviewer 1: Results
Lines 98-101. These results seems unexpected. I would expect greater variation between geographic populations that, presumably, have low (negligible) gene flow between populations.

Reply: we previously demonstrated (Manni et al 2015; 2017) that the geographic proximity of populations does not affect the genetic connectivity as a consequence of the chaotic dispersion pattern of this mosquito. The rapid invasion pattern was not the consequence of a step by step process, but it was the consequence of multiple introductions, high propagule pressure, admixture and adaptive life history traits (lines 53-54). This allows the establishment and persistence of introduced populations. Admixture generated novel genetic combinations allowing populations to establish. In line with this, the AMOVA, data suggest that the overall divergence is mainly due to the molecular differences among individuals. Moreover, the fact that the variance among regions is low suggests that the geographic proximity among populations does not affect the genetic connectivity among populations.
Note that *Ae. albopictus* took 4 decades to invade the globe, while *Ae. aegypti* did so in 4 centuries (lines 48-49).

Reviewer 1: Lines 169-171. *Aedes albopictus* was first recognized to establish a persistent population in Texas.
The description of relationships and origins of populations seem adequate.

Lines 207-209. The baseline high levels of disseminated infection hamper the ability to observe genetic factors may enhance disseminated infection. In contrast, the ability to assess genetic-associated reductions in disseminated infection is feasible.

<u>Discussion</u>
Overall, this section is well written.
Lines 359-363. Susceptibility to infection was not measured in this study (i.e., midgut infection barrier(s)).

Reply: Many thanks for this comment. The sentences from the mentioned lines were rephrased and midgut was replaced by “barriers for CHIKV dissemination (i.e. antiviral immune responses at the midgut epithelium)”.

Reviewer 1: Lines 391-393. Rephrase for clarity.
It would be helpful to describe limitations of vector competence measurements in determining a mosquito's ability to transmit a pathogen. That is, several other factors interact to determine the probability of an infectious bite is received by a susceptible host.

Reply: We should precise that beyond the differences on transmission efficiencies mentioned in this sentence, unneglectable differences of extrinsic incubation period were also detected at low temperatures between Asian and ECSA strains. The sentence has been rephrased, with references added to mention this information and the possible influence of other vectorial capacity parameters:
"It has been shown that exposure of European *Ae. albopictus* to low temperatures (20°C) significantly increased the extrinsic incubation period and reduced the transmission efficiency of Asian CHIKV strains, while for strains from ECSA lineages these parameters remained unaffected. Although other factors influencing vectorial capacity may interact and determine that an infectious bite is received by a susceptible human host, these results firstly highlight the importance of three-way interactions between temperature, the mosquito population and the viral genotype..."

Reviewer 1: <u>Material and Methods</u>
Lines 471-473. Please provide information on the numbers of founding individuals from the field that were used to establish laboratory colonies. According to Table 1, very few founding individuals comprised the establishing laboratory population. This approach brings into question whether populations went through a bottleneck which may result in founder effects.

Reply: I think there has been a misunderstanding, as mosquito populations used for vector competence estimations were generated from 15 to 17 ovitraps/site of collection (so we obtained about 600-1000 individuals per population). Among those, thirty, on average, were provided for microsatellite analyses. The numbers in Table 1 only mentioned the mosquitoes used for microsatellite analyses so we added this precision for clarity on the table and we added the information on the founding individuals for vector competence in the Mosquito collection section. So according to our sampling strategy, there should not be a founder effect for the mosquito populations used for vector competence assays and for SSR genotyping.

Reviewer 1: Please specify the generation tested for the genetic and vector competence assays. Many collections were made in 2010-2013. Depending on when the tests were performed, these mosquitoes have been maintained in laboratory conditions for numerous generations and may not be representative of field populations.

Reply: the microsatellite genotyping analyses were based on an average of 30 G0 individuals for each population and we used the corresponding G1 generation for vector competence assays except for Brazzaville (G2) and Cesena (G3) populations. This information was added in the section "Mosquito collection" in materials and methods. In laboratory tests we found no difference in vector competence from F1 to F3, depending on the number of progenitors.

Reviewer 1: Lines 478-480. This approach seems like a good strategy to address the focus of the study.

Lines 492-494. It seems appropriate to cite the following paper to support this statement; Chen, X-G, et al. 2015. Genome sequence of the Asian tiger mosquito *Aedes albopictus*, reveals insights into its biology, genetics, and evolution. Proc Natl Acad Sci USA 112(44): E5907-E5915.

Reply: The citation has been added

Reviewer 1: Line 501. "…well-documented…"

Reply: we have added a reference

Reviewer 1: The genetic diversity analyses, population structure, and analyses of demographic history are appropriate and comprehensive.

Lines 574-576. This explanation is helpful to the reader.

Line 587. Please provide additional details on how the vector competence experiments were performed (e.g., how was saliva collected?). When were mosquitoes tested for disseminated infection and saliva infection? Were mosquitoes tested at more than one time period following ingestion of CHIKV infected blood?

What was the viral titer in the infected bloodmeal? Was it representative of viremia in humans. The viral dose will influence disseminated infection and saliva infection.

Reply: All information regarding viral titre, days post infections tested, viral strains etc are already in S4 Table, as mentioned in the "Vector competence data for CHIKV strains" and in the corresponding result section. The protocol used for salivation was the following: the wings and legs were removed from each mosquito and the proboscis was inserted into a 20-µl tip containing 5µl of FBS. After 45 min of salivation, FBS containing saliva was expelled into 45µl of Leibovitz L15 medium for titration. We added the corresponding reference for salivation protocol in the text.

CHIKV infections are characterized by high viremia levels (10^4 to 10^8 RNA copies/mL (Laurent et al., Clin. Chem. 53 (8), 1408–1414 (2007); Lanciotti et al., Emerg Infect Dis. 13(5) (2007)). The titre of infectious blood meals was $10^{6.5}$ – $10^{7.5}$ pfu/mL.

Reviewer 1: Lines 588-589. Please specify generation number for each population.

Reply: This information has been added in the "Mosquito collection" section.

Reviewer 1: Lines 594-596. Transmission efficiency suggests the occurrence of a transmission event (e.g., passage of pathogen to a susceptible vertebrate host). Saliva infection (or transmission potential) seems like a better descriptor.

Reply: Renaming the vector competence estimators used could be misleading for readers as in this paper we conducted statistical analysis using data that were presented as "transmission efficiencies" in previous studies. We believe that it is more accurate (and clearer for the readers that want to consult those previous studies for additional information) to use in our paper the same name for the different vector competence estimators used. The definition provided for "transmission efficiency" in the manuscript is

commonly used in vector competence studies and clearly shows that the transmission to a susceptible vertebrate host is not assessed.

Reviewer #2 (Remarks to the Author):

Reviewer 2: This paper reports an analysis of 11 microsatellites that have been genotyped in a large collection of mosquitoes. This is used to reconstruct the dispersal of the mosquitoes round the world. The vector competence of mosquitoes was then measured and linked to the genetic data. Given the importance of this species these results are of general interest, and the link to vector competence was novel.

A particularly curious feature of the paper is that you can read the abstract, introduction, results, discussion and conclusion without knowing what data is being analysed. In an era when large genomic datasets are common, this left me wondering whether this was intentional. Regardless, it makes it very hard to understand the results. The fact that this is an analysis of 11 microsatellite loci should be stated in the abstract and the first line of the results.

Reply: We introduced this information in the SUMMARY and in the RESULTS sections. We had not mentioned the use of SSRs as markers as this information was available in the material and Methods section where we explained that their use allowed us to perform accurate biogeographic analyses at the macrogeographic scale (Pless et al. 2017). SSRs provide a reliable resource for ABC simulations (Benazzo et al. 2015). They represent a good balance between being well characterized (i.e. in terms of mutation model) and having sufficient genetic diversity to provide statistical power, as we previously assessed (Manni et al. 2015). Moreover our SSRs are spread across the genome as we assessed (see Materials and Methods) as such they may not be affected by selection or other factors that impact the data.

Reviewer 2: The most novel aspect of this work was to link the demographic history of populations to their vector competence. The analysis that was performed appears to show little more than that there is geographical variation in vector competence.

Reply: Yes, we demonstrated that there is population geographical variation in competence for CHIKV. BUT, most importantly, we demonstrate that the population geographical variation in competence for the different CHIKV genotypes, is strongly dependent on the degree of ancestry of populations within one of the genetic lineages that historically diversified and diverged during the recent mosquito invasion process. This is a novelty.

Reviewer 2: As nearby populations tend to be genetically similar, any geographical structure in phenotype will correlate with genotype.
Geographical structure could arise for reasons other than shared ancestry, such as adaptation to local conditions. What would be considerably more interesting would be to show that genetic ancestry predicts vector competence after controlling for geography. This could easily be achieved by adding a measure of geographical proximity to the statistical model. I am sure there are more elegant ways to approach this

Reply: We previously demonstrated, using different markers and a greater number (23) of SSRs (Manni et al 2015; 2017) that the population geographic proximity of populations does not affect the genetic connectivity as a consequence of the chaotic dispersion pattern of this mosquito. In this paper AMOVA analyses (Lines 98-101) showed that only 6% of variance was detected between regions confirming that geographic proximity does not impact the population genetic connectivity. Secondly, no signs of isolation by distance (IBD) were detected globally (y = 1e-06x + 0.0805; R^2 = 0.0149) nor within regions. We added this information in the text. More importantly, lineages may span across continents (e.g. *K*2, *K*4, *K*5, *K*6, see Figures 2 and 5 and Table 2). By itself, this data already tells us that geography is not a factor in shaping the genetic structure of populations around the world. For example, in the Mediterranean area different lineages are present, despite the geographic population proximity.
The reasons why we hypothesized that the degree of ancestry among the populations affects the degree of competence, is explained in the text (lines 80-84 and the references included). Our analysis shows that "ancestry is associated to vector competence"

Reviewer 2: The way that the ancestry components were included in the analysis of viral titres seems rather adhoc. Why consider just one or two of the components. I can see no reason why this is done. I would suggest that the 6 ancesents are added to the model as fixed effects. It would still be possible to test the overall significance of genetic ancestry. I found the description of these statistics in the methods hard to follow. Please add a formal equation that describes the model used.

Reply: I think there is a misunderstanding as the 6 *K*-ancestry components were considered in the analysis. We added information in the Results and Material and Methods sections to clarify the strategy used for the analyses.

Reviewer 2: The interpretation of the ancestry fractions is very literal, despite this species clearly having undergone changes in population size that are known to confound these analyses. These issues are described in a paper by Daniel Falush (A tutorial on how not to over-interpret STRUCTURE and ADMIXTURE bar plots, Nature Communication), and their effects in a related species of mosquito are discussed in Crawford et al, BMC Biology. Throughout the manuscript the literal interpretation of these plots as a set of ancestral populations should be avoided in the absence of supporting information. This may require rewriting significant sections of text.

Reply: In this paper we provided "a picture of Major events which determined at macrogeographic level the demographic histories of populations" (see lines 84-85). To do this we derived data NOT ONLY from STRUCTURE and ABC analysis but also from Historical and demographic information available on the invasive process across the species range. We used this information in the Discussion section.
The dynamics of "ancestry fractions" and lineages across the considered areas, as presented in Table 2, are clearly related to the historical records of invasion processes, as clearly shown in Figure 2. So, our interpretation of "fraction ancestry" is not merely "literal"
The historical lineage diversifications occurred through admixture events among different genetic compositions (lines 276-277). Multiple introductions, propagule pressure, admixture and adaptive life history traits (lines53-54) were key features for the establishment and persistence of introduced populations buffering the problems of

bottleneck effects. Admixture generated novel genetic combinations allowing populations to establish, further supporting the role of multiple introductions in promoting ‘invasiveness’ (Manni et al 2015; 2017; Cunze 2018; Sherpa et al 2019). Note that *Ae. albopictus* took 4 decades to invade the globe, while *Ae. aegypti* did so in 4 centuries (lines 48-49).
The paper by Crawford *et al*. shows how inferring migration / admixture and even foundation events from ancestry data can be misleading. In the paper, demographic models were ultimately used to explain such misinterpretations. The paper by Lawson, van Dorp and Falush warns against naïve inferences and over-interpretations of STRUCTURE and ADMIXTURE. Again, our conclusions are not based solely on the STRUCTURE data, but we considered the ABC results as well (which are more detailed regarding the demographic dynamics) and demographic data.

Reviewer 2: The ABC analyses are the second approach taken to demographic reconstruction. In the main text there is no indication of statistical uncertainty, which made it very hard to interpret these results. The supplementary table suggests that many of the relationships are well supported (they are found across all the well-supported models). It would be helpful to show this information on the figure in some way to highlight poorly supported relationships.

Reply:
We introduced the confidence intervals in Figure 3.

Reviewer 2: A second source of uncertainty is the long list of assumptions being made – priors, changes in Ne, mutation rates etc. It is very hard to understand if altering these alters the outcome. It would be helpful to explore some of these issues, perhaps with a supplementary results that repeat the analyses with different assumptions.
This, together with the comments in the preceding paragraph, left me uncertain how robust the scenario in the summary figure is.

Reply:
The long list of assumptions in S7 table were the outcome of previously chosen and tested assumptions in the previous paper on ABC by Manni *et al* 2017. That these assumptions are robust derives by the fact that when we re-tested the same scenarios considered by Manni et al (under these assumptions), as described in the Results section, we obtained the same results also when we included new populations for which we had to test the derivation.

Reviewer 2: The prose in the results section was hard to read and follow as it tended to involve a very detailed repetition of information in figures and tables. To understand the major conclusions, I found it simpler to look at the images. I would suggest simplifying the text.

Reply:
As suggested, we have attempted to simplify the text, where possible

Reviewer 2: A few minor comments:
The word ‘variation’ should not be used to replace ‘variance’ (eg line 99). The former has no precise statistical meaning.

Reply: Many thanks for the comment. It has been done.

Reviewer 2: I struggled to follow much of the first paragraph which is largely describing Fst. For example, this sentence 'The most homogeneous area was the ancestral Southeast Asia, with populations that maintained low levels of differentiation with La Réunion, the Mediterranean Basin, Hawaii and North America' homogenous needs defining (Fst I guess), and some numbers put on how this conclusion is reached.
The density of geographical sampling seems to affect this result. Similarly, 'Levels of structuring were observed within La Réunion, while the Mediterranean area appears to be heterogeneous.' Means little to me. Fst within le reunion looks rather low to me. Overall, this paragraph could do with simplifying to get to the take-home message – the detailed description of the figure seemed less informative than looking at the picture

Reply:
F_{ST} estimates provided in Table S2, have been introduced into the text. Homogeneous is intended in terms of F_{ST} (non-significant or very, very low). Some simplifications were introduced into text. I agree that the description of this table can be tedious, but it can give the keys to support and interpret the ancestry profiles of populations that are described later. For instance, Asia which maintains low F_{ST} with La Réunion and the Mediterranean area etc is in *K*6 with these populations.

Reviewer 2: Maybe Table 2 could go as a supplement? Fig 2 seems to convey this information more effectively

Reply: We think it is important to maintain it into the text as it helps readers to interpret the dynamic of lineage diversifications and this table also helps interpretation of the results related to association of "ancestry fractions ".

Reviewer 2: Lines 123-139 again describes a lot of results. I was left looking at the figure in order to conclude the results are really rather interesting. Some rewriting could make the prose much more impactful – scrap the names of ancestry components and other details that are all in Tables and Figures, and instead lead with the biological insight. The different ancestry of relatively nearby populations is very striking.

Reply: The different ancestry of nearby populations is expected considering that the invasion was not step by step but in a chaotic manner. In fact, we did not find isolation by distance but still, the data of ancestry fractions help the reader to better understand the results related to the association "fraction of ancestry" and competence.

Reviewer 2: Define abbreviations at first use (eg DE). Reducing the use of abbreviations aids readability.

Reply: It has been done

Reviewer 2: Line 207. It reads very oddly not to mention what the experiment was here. There seems to be a presumption the methods are read first.

Reply: We have added the appropriate information into the results section for clarity.

REVIEWERS' COMMENTS:

Reviewer #1 (Remarks to the Author):

Previously, I provided a comprehensive review of the originally submitted paper.

I have now had a chance to review the rebuttal letter, revised paper, supplemental files, as well as the marked-up version of the original paper.

The authors did a good job at addressing the reviewer concerns.

The experimental design and statistical analyses are appropriate, and the findings are convincing.

I am satisfied with the current version and have no additional requested edits.

Best regards.
